# Inner membrane YfgM–PpiD heterodimer acts as a functional unit that associates with the SecY/E/G translocon and promotes protein translocation

**DOI:** 10.1016/j.jbc.2022.102572

**Published:** 2022-10-07

**Authors:** Ryoji Miyazaki, Mengting Ai, Natsuko Tanaka, Takehiro Suzuki, Naoshi Dhomae, Tomoya Tsukazaki, Yoshinori Akiyama, Hiroyuki Mori

**Affiliations:** 1 Division of Biological Science, Graduate School of Science and Technology, Nara Institute of Science and Technology, Ikoma, Japan; 2 Institute for Life and Medical Sciences, Kyoto University, Kyoto, Japan; 3 Biomolecular Characterization Unit, RIKEN Center for Sustainable Resource Science, Saitama, Japan

**Keywords:** membrane protein, molecular chaperone, protein complex, protein crosslinking, protein secretion, *p*BPA, SecD/F, VemP, tetratricopeptide repeat domain, AlphaFold2, BAM, β-barrel assembly machinery, FL, full length, HRP, horseradish peroxidase, IM, inner membrane, MBP, maltose-binding protein, MS, mass spectrometry, *p*BPA, *p*-benzoyl-l-phenylalanine, PBST, PBS with Tween-20, Sec, SecY/E/G, Spc, spectinomycin, TM, transmembrane, TPR, tetratricopeptide repeat, TTAC, translocation-coupled translation arrest-cancellation, OMP, outer membrane protein, VemP, *Vibrio* protein export monitoring polypeptide

## Abstract

PpiD and YfgM are inner membrane proteins that are both composed of an N-terminal transmembrane segment and a C-terminal periplasmic domain. *Escherichia coli* YfgM and PpiD form a stable complex that interacts with the SecY/E/G (Sec) translocon, a channel that allows protein translocation across the cytoplasmic membrane. Although PpiD is known to function in protein translocation, the functional significance of PpiD–YfgM complex formation as well as the molecular mechanisms of PpiD–YfgM and PpiD/YfgM–Sec translocon interactions remain unclear. Here, we conducted genetic and biochemical studies using *yfgM* and *ppiD* mutants and demonstrated that a lack of YfgM caused partial PpiD degradation at its C-terminal region and hindered the membrane translocation of *Vibrio* protein export monitoring polypeptide (VemP), a *Vibrio* secretory protein, in both *E. coli* and *Vibrio alginolyticus*. While *ppiD* disruption also impaired VemP translocation, we found that the *yfgM* and *ppiD* double deletion exhibited no additive or synergistic effects. Together, these results strongly suggest that both PpiD and YfgM are required for efficient VemP translocation. Furthermore, our site-directed *in vivo* photocrosslinking analysis revealed that the tetratricopeptide repeat domain of YfgM and a conserved structural domain (NC domain) in PpiD interact with each other and that YfgM, like PpiD, directly interacts with the SecG translocon subunit. Crosslinking analysis also suggested that PpiD–YfgM complex formation is required for these proteins to interact with SecG. In summary, we propose that PpiD and YfgM form a functional unit that stimulates protein translocation by facilitating their proper interactions with the Sec translocon.

In gram-negative bacteria, around 30% of proteins are localized and function at the cell surface on the inner membrane (IM), periplasm, or outer membrane. After being synthesized in the cytoplasm, these proteins must be translocated across and/or integrated within the IM to reach their destination. The evolutionally conserved SecY/E/G (Sec) translocon, which consists of three integral IM proteins (SecY, SecE, and SecG), creates a narrow channel for the membrane translocation of newly synthesized polypeptides (1, 2, 3, 4). SecY, the central component of the translocon, contains 10 transmembrane (TM) segments that form the protein translocation channel (2, 5), whereas SecE and SecG peripherally associate with SecY (2, 4). Since the Sec translocon is a passive channel, an essential ATPase motor (SecA) (6, 7, 8) and an IM-integrated proton motive force–driven motor (SecD/F complex) (9, 10) drive polypeptide movement through the Sec translocon from the cytoplasmic and periplasmic sides, respectively. In addition, the Sec translocon can interact with the membrane protein insertase, YidC, to integrate membrane proteins into the IM (11, 12, 13, 14). It has been proposed that membrane protein TM segments are transferred from the Sec translocon into the lipid phase of the IM *via* the lateral gate formed between TM2 and TM7 of SecY (1, 15). In contrast to a wealth of accumulated knowledge about structure and function of the Sec translocon and the motor proteins, only a limited information regarding functional relationships between these Sec components and other Sec-related factors that could contribute to maturation process of secretory proteins is available.

During and/or after the translocation of proteins through the Sec translocon, the proper folding of periplasmic proteins and the efficient targeting of outer membrane proteins (OMPs) are regulated by many periplasmic chaperones, including SurA (a peptidyl–prolyl *cis–trans* isomerase, PPIase), Skp, DegP, and PpiD (16, 17, 18). Among these chaperones, PpiD is unique in that it is a membrane protein that associates with the IM *via* a single N-terminal TM segment, whereas its large periplasmic C-terminal domain contains a parvulin-like PPIase domain, similar to SurA (19). The *ppiD* gene was first identified as a multicopy suppressor of a *surA* null mutation, and it was reported that *ppiD* deletion significantly decreases cellular OMP levels, whereas the *ppiD* null mutation causes synthetic lethality with the *surA* null mutation (19). It was therefore concluded that PpiD plays an important role in OMP biogenesis; however, several later studies were unable to reproduce published data supporting the functional importance of PpiD in OMP biogenesis (20, 21, 22). These studies instead reported genetic and physical interactions between PpiD and other periplasmic chaperones (SurA, Skp, and DegP), suggesting that PpiD participates in the periplasmic chaperone network and functions mainly to facilitate the early periplasmic folding of newly translocated proteins (21, 22).

*In vitro* studies have also suggested that PpiD plays a role in protein translocation mediated by the Sec translocon by interacting with SecY/E/G and nascent polypeptides emerging from the translocon (23). Detailed photocrosslinking analyses have suggested that PpiD contacts the lateral gate region of SecY (24) and that the periplasmic domain of PpiD is located close to SecG (25). Recently, we used the pulse-chase and *in vivo* photocrosslinking experiment (PiXie) method (26) to demonstrate that PpiD interacts directly with the nascent translocating *Vibrio* protein export monitoring polypeptide (VemP) (27), which undergoes regulated translation elongation arrest in response to decreased cellular protein translocation activity (28). Our *in vivo* studies have also indicated that physical interaction and cooperation between PpiD and SecD/F are required for the efficient translocation and translation arrest-cancellation of VemP. Based on these observations, we proposed that PpiD stimulates the forward movement of polypeptide substrates through the Sec translocon by capturing the substrate and transferring it to SecD/F and/or other periplasmic chaperones during the later stages of translocation (28).

YfgM and PpiD share the same type II (N_IN_–C_OUT_) topology; they integrate into the IM *via* N-terminal TM segments, whereas the large C-terminal domain is exposed to the periplasm. The YfgM periplasmic domain is composed of four tetratricopeptide repeat (TPR) motifs, and TPR motif–containing domains (TPR domains) generally act as scaffolds to mediate protein–protein interactions (29). Several studies have suggested that PpiD forms a stable complex with YfgM (30, 31, 32) that interacts with the Sec translocon (30), indicating that the function of YfgM is related to that of PpiD. Although *yfgM* gene deletion has been shown to induce cell envelope stress responses (30), the physiological role of YfgM in protein translocation remains unclear, and the physiological significance and the molecular basis of the PpiD–YfgM interaction are unresolved.

In this study, we conducted genetic and biochemical studies using *yfgM* and *ppiD* mutant strains and found that YfgM stabilizes PpiD and is involved in VemP translocation. In addition, we investigated the modes of interaction between YfgM and PpiD and the Sec translocon through *p*-benzoyl-l-phenylalanine (*p*BPA)-mediated *in vivo* photocrosslinking analyses (33, 34) targeted to YfgM, PpiD, and SecG. Based on these results, we discuss the possible physiological significance of YfgM–PpiD complex formation.

## Results

### YfgM stabilizes the partner protein PpiD

**Figure 1:**
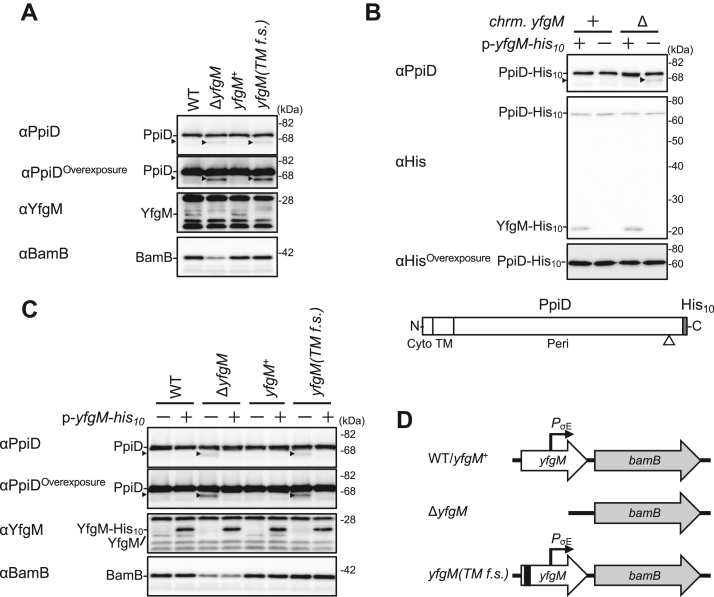
**YfgM stabilizes its partner protein PpiD.***A*, generation of PpiD degradation products in *yfgM* mutant cells. *yfgM(TM f.s.)* represents a mutant strain expressing a YfgM derivative in which the original TM segment had been replaced by an unrelated amino acid sequence as a result of frameshift mutations. NT17 derivative cells were grown at 30 °C in L-medium for 2.5 h. Total cellular proteins were acid-precipitated and analyzed using SDS-PAGE and immunoblotting with the indicated antibodies. *B*, PpiD degradation occurred near its C terminus in the absence of YfgM. HM5894 (*ppiD-his*_*10*_) and HM5895 (*ppiD-his*_*10*_, Δ*yfgM*) cells carrying pMW118 or pMW118-*yfgM-his*_*10*_ were grown at 30 °C in L-medium until early log phase and induced with 1 mM IPTG for 1 h. Total cellular proteins were acid-precipitated and analyzed using SDS-PAGE and immunoblotting with the indicated antibodies. A schematic diagram of PpiD-His_10_ is provided in which the approximate position of the cleavage site is indicated as an *open arrowhead*. *C*, effects of YfgM overexpression in *yfgM* mutant cells. NT17 derivative cells carrying pMW118 or pMW118-*yfgM-his*_*10*_ were grown at 30 °C in L-medium until early log phase and induced with 1 mM IPTG for 1 h. Total cellular proteins were acid-precipitated and analyzed using SDS-PAGE and immunoblotting with the indicated antibodies. *Black arrowheads* in (*A*–*C*) indicate PpiD degradation products (PpiD′). *D*, schematic diagrams of the *yfgM* and *bamB* genes in wildtype and *yfgM* mutant cells. TM, transmembrane.

Previous studies have shown that YfgM forms a stable complex with PpiD (30, 31, 32). Therefore, we first examined whether YfgM affects the stability of PpiD in *Escherichia coli* using a strain lacking the *yfgM* gene. Immunoblot analyses of total cellular proteins using anti-PpiD antibodies showed that the absence of YfgM exerted no detectable effect on the cellular levels of the full-length (FL) PpiD protein, as reported previously (Fig. 1*A*, *top panel*; (30)). However, we noticed that a faint band (hereafter denoted as PpiD′) migrated slightly faster than FL PpiD in the Δ*yfgM* strain (Fig. 1*A*, *top and upper-middle panels*). To examine whether PpiD′ was derived from FL PpiD, we monitored the stability of fully synthesized PpiD after blocking cellular *de novo* protein synthesis using spectinomycin (Spc; Spc-chase experiment). The amount of PpiD′ gradually increased with a concomitant decrease in the levels of FL PpiD during the Spc-chase period (Fig. S1), strongly suggesting that PpiD′ is a degradation product of FL PpiD. In cells chromosomally expressing a PpiD derivative with a C-terminal His_10_-tag (PpiD-His_10_), the degradation product was detected with anti-PpiD but not with anti-His antibodies, even after prolonged exposure during immunoblot visualization (Fig. 1*B*, *bottom panel*). In addition, ectopic YfgM-His_10_ expression in the Δ*yfgM* strain suppressed the generation of PpiD’ (Fig. 1, *B* and *C*). Taken together, these results suggest that a C-terminal portion of PpiD becomes susceptible to degradation by one or more unidentified proteases in the absence of YfgM (Fig. 1*B*, *schematic picture*).

It has been reported that the *yfgM* gene contains a σ^E^-dependent promotor for the downstream *bamB* gene, which encodes a component of the β-barrel assembly machinery (BAM) complex that is crucial for OMP biogenesis (Fig. 1*D*) (35). Consistently, we found that BamB accumulation levels were appreciably lower in our *yfgM* deletion strain than in the wildtype strain (Fig. 1*A*, *bottom panel*), indicating that the observed decrease in BamB levels is somehow involved in PpiD′ generation through the perturbation of OMP biogenesis. Therefore, we constructed a *yfgM* mutant strain carrying a mutant gene (*yfgM(TM f.s.)*) that expresses a nonfunctional YfgM variant (YfgM(TM F.S.)) but retains the intact σ^E^-dependent promotor (Fig. 1*D*). Within the YfgM(TM F.S.) protein, the hydrophobic amino acid sequence for the TM segment of YfgM (Ala-24 to Asn-43) was replaced by a completely different hydrophilic sequence because of frameshift mutations (Fig. S2). Consequently, YfgM(TM F.S.) did not accumulate in cells, likely as it was not targeted to the IM (Fig. 1*A*, *lower-middle panel*). Although BamB accumulation was comparable in the *yfgM(TM f.s.)* and wildtype strains, as expected (Fig. 1*A*, *bottom panel*), PpiD′ was still detected as in the Δ*yfgM* strain (Fig. 1*A*) and YfgM-His_10_ overproduction from a plasmid suppressed PpiD′ generation (Fig. 1*C*). These results indicate that the absence of YfgM is the main reason for the generation of PpiD′ and strongly suggest that YfgM stabilizes PpiD, presumably by forming a YfgM–PpiD complex.

### YfgM acts coordinately with PpiD in the translocation-coupled translation arrest-cancellation of VemP

**Figure 2:**
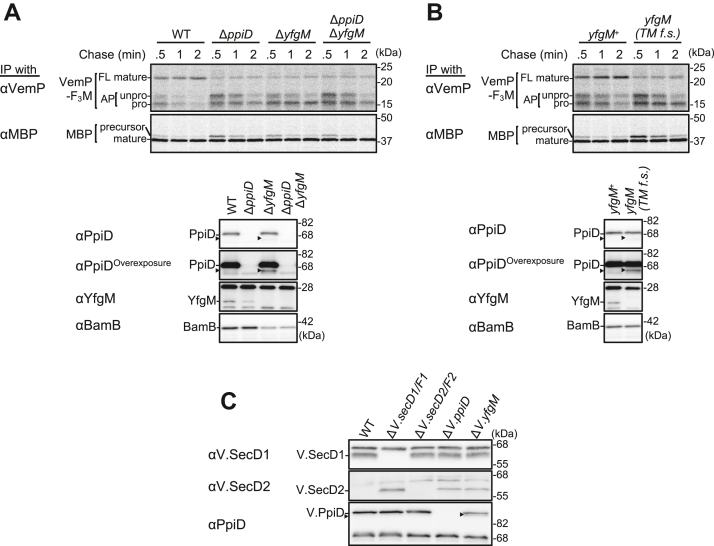
**YfgM and PpiD are involved in the translocation-coupled translation arrest-cancellation of VemP.***A* and *B*, effects of *yfgM* mutations on the stability of arrested VemP. *Upper*: NT17 derivative cells with the indicated mutation carrying both pSTD343(*lacI*) and pHM1021-*vemP-f*_*3*_*m* were grown at 30 °C in M9-based medium until early log phase, induced with 1 mM IPTG for 15 min, pulse-labeled with [^35^S]Met for 30 s, and chased for the indicated periods. At each time point, total cellular proteins were acid-precipitated, subjected to IP with anti-VemP or anti-MBP antibodies, and analyzed using SDS-PAGE followed by phosphorimaging. *Lower*: Before RI labeling, total cellular proteins from some cells were acid-precipitated and analyzed using SDS-PAGE and immunoblotting with the indicated antibodies. *Black arrowheads* in (*A* and *B*) indicate PpiD degradation products (PpiD′). *C*, effects of *yfgM* deletion on *V.secD2* expression in *Vibrio* cells. *Left*: The indicated *Vibrio* cells were grown at 30 °C in VC medium for 2 h. Total cellular proteins were acid-precipitated and analyzed using SDS-PAGE and immunoblotting with the indicated antibodies. *Black arrowheads* represent presumed V.PpiD degradation products. IP, immunoprecipitation; MBP, maltose-binding protein.

Since PpiD, the partner protein of YfgM, plays a crucial role in the translocation-coupled translation arrest-cancellation (TTAC) of VemP (28) and the PpiD–YfgM complex is known to interact with the Sec translocon (30), we next investigated the possible role of YfgM in the maturation (translocation) of VemP. To this end, we examined the effect of *yfgM* deletion on the TTAC kinetics of VemP using a model substrate, VemP-3xFLAG-Myc (VemP-F_3_M; a VemP derivative containing a 3xFLAG tag and a Myc tag at its C terminus). The addition of these tags allowed the three VemP-derived species to be discriminated using SDS-PAGE: the arrested product with an unprocessed signal sequence (AP-unpro), the arrested product lacking the signal sequence (AP-pro), and the FL mature product (FL-mature; Fig. 2*A*) (36). We expressed VemP-F_3_M from a plasmid in *E. coli* and examined its behavior using pulse-chase experiments. Consistent with previous results (36), the arrested VemP products (AP-unpro and AP-pro) were gradually converted into FL-mature in the wildtype strain, whereas this process was obviously retarded in the *ppiD* and *yfgM* deletion strains, indicating that the TTAC of VemP was slowed (Figs. 2*A* and S3*A*). The translation-arrested VemP state was also stabilized in the *yfgM(TM f.s.)* mutant strain (Figs. 2*B* and S3*B*), and YfgM expression from a plasmid canceled the stabilization of the arrested VemP state in the *yfgM* deletion strain (Fig. S4). Importantly, the Δ*yfgM* mutation had no additive or synergistic effect on the stabilization of the arrested VemP state (Figs. 2*A* and S3*A*) when introduced into the Δ*ppiD* mutant strain, suggesting that YfgM and PpiD act at the same step. By contrast, all these mutations examined only slightly retarded signal sequence cleavage of maltose-binding protein (MBP), a Sec-dependent secretory protein (Figs. 2, *A*, *B*, S3, *A*, and *B*), showing that both PpiD and YfgM do not play crucial roles in the initial step of the MBP export before its signal peptide processing.

The effects of *ppiD* and *yfgM* deletion on the TTAC of VemP were confirmed using a VemP–PhoA reporter assay (Fig. S5*A*). The wildtype strain with a plasmid carrying the *vemP–phoA* fusion gene displayed significant PhoA activity as the FL VemP–PhoA fusion protein was translated and exported into the periplasmic space because of effective VemP arrest-cancellation in this strain. Conversely, the relative PhoA activity in the Δ*yfgM* and the Δ*ppiD* mutant strains expressing VemP–PhoA was about half of that in the wildtype strain expressing VemP–PhoA, suggesting that the translation-arrested VemP state was more stabilized in these mutant strains than in the wildtype strain. Again, no additive or synergistic effects were observed in the double mutant strain. Together, these results indicate that YfgM and PpiD likely affect the TTAC of VemP by forming a functional complex.

Since VemP monitors cellular protein export activity and regulates *V.secD2/F2* gene expression *via* translation arrest in *Vibrio alginolyticus* (27), we examined the effect of *yfgM* deletion on VemP translation-arrest–mediated V.SecD2 expression in *V. alginolyticus*. In Na^+^-rich medium, V.SecD2 expression was strongly repressed because V.SecD1/F1 was fully functional, leading to efficient VemP arrest-cancellation (27). In contrast, V.SecD2 accumulation increased in the *yfgM* deletion *V. alginolyticus* strain as well as in the Δ*V.secD1/F1* and Δ*V.ppiD* strains (Fig. 2*C*, *middle panel*) (27). In addition, a faster-migrating band similar to *E. coli* PpiD′, presumably representing a V.PpiD degradation product, was detected in the Δ*V.yfgM* strain (Fig. 2*C*, *bottom panel*). These results suggest that YfgM plays a role alongside PpiD in VemP translation arrest-cancellation and V.SecD2 repression under Na^+^-rich conditions in *Vibrio* cells.

### Modes of YfgM–PpiD and YfgM–Sec translocon interactions *in vivo*

**Figure 3:**
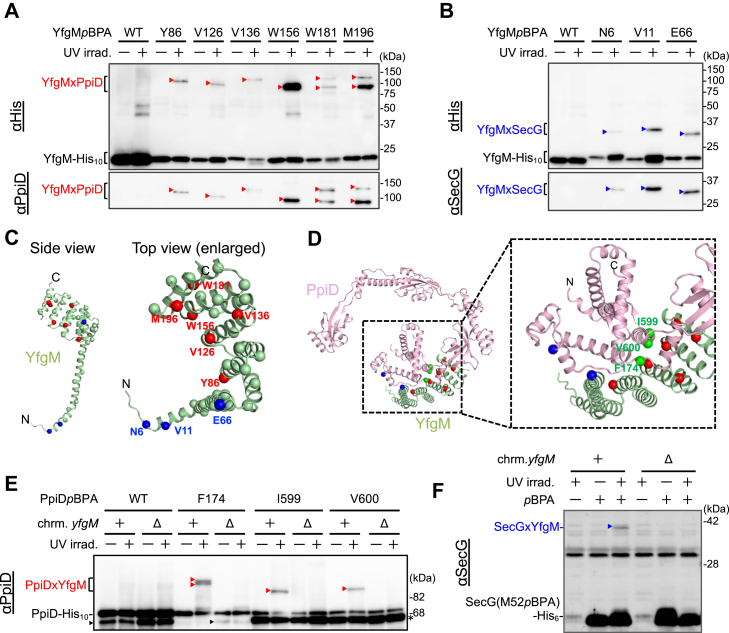
**YfgM interacts with PpiD and the Sec translocon *in vivo*.***A* and *B*, *in vivo* photocrosslinking analysis of YfgM. NT35 (Δ*yfgM*) cells carrying both pEVOL-pBpF and pMW118-*yfgM(amb)-his*_*10*_ were grown at 30 °C in L-medium containing 0.5 mM *p*BPA until early log phase and induced with 1 mM IPTG for 1 h to express the indicated YfgM(*p*BPA) variants. Cultures were divided into two portions treated with or without UV irradiation for 10 min at 4 °C. Total cellular proteins were subjected to pull-down with nickel–nitrilotriacetic acid agarose before being analyzed using SDS-PAGE and immunoblotting with the indicated antibodies. *C*, mapping of PpiD-/SecG-crosslinking residues of YfgM onto its structural model predicted by AlphaFold2. *p*BPA-incorporated sites are indicated by *spheres*. PpiD and SecG crosslinking sites are indicated in *red* and *blue*, respectively. *D*, mapping of PpiD and YfgM crosslinking residues onto the structural model of the PpiD–YfgM complex predicted by AlphaFold2. *p*BPA-incorporated sites in PpiD are indicated by *green spheres*. PpiD-crosslinking sites in YfgM are indicated by *red spheres*. SecG-crosslinking sites in PpiD (F122) and YfgM (E66) are indicated by *blue spheres*. *E*, *in vivo* photocrosslinking analysis of PpiD. RM3688 (Δ*ppiD*) or RM3690 (Δ*ppiD*, Δ*yfgM*) cells carrying both pEVOL-pBpF and pHM1021-*ppiD(amb)-his*_*10*_ were grown at 30 °C in L-medium containing 0.5 mM *p*BPA and 0.02% arabinose until early log phase and induced with 1 mM IPTG for 1 h to express the indicated PpiD(*p*BPA) variants. Cultures were divided into two portions treated with or without UV irradiation for 10 min at 4 °C. Total cellular proteins were acid-precipitated and analyzed using SDS-PAGE and immunoblotting with the indicated antibodies. *Black arrowheads* indicate PpiD degradation products (PpiD′) that should be produced from uncomplexed (YfgM-free) PpiD. Some PpiD′ was generated even in the *yfgM*^+^ strain (first and second lanes), likely because PpiD overproduction from a plasmid results in YfgM-free PpiD accumulation. *Bands* (marked with an *asterisk*) slightly smaller than full-length PpiD-His_10_ in the PpiD(I599*p*BPA) and PpiD(V600*p*BPA) lanes likely represent the amber fragments of PpiD and/or the degradation products of PpiD derivatives (PpiD′). *F*, *in vivo* photocrosslinking analysis of SecG. NT23 (Δ*secG*) or NT31 (Δ*secG*, Δ*yfgM*) cells carrying both pHM649 and pMW118-*secG(M52amb)-his*_*6*_ were grown at 30 °C in L-medium with 0.5 mM *p*BPA until midlog phase. Cultures were divided into two portions treated with or without UV irradiation for 10 min at 4 °C. Total membrane proteins were analyzed using SDS-PAGE and immunoblotting with anti-SecG antibodies.

Although YfgM is known to interact with PpiD to form a stable complex that interacts with the Sec translocon (30), the molecular details of these interactions are currently unknown; therefore, we conducted a systematic photocrosslinking analysis to elucidate how YfgM interacts with PpiD and SecY/E/G. We constructed 40 different YfgM(*p*BPA)-His_10_ derivatives by systematically introducing *p*BPA into every fifth residue in YfgM. Total cellular proteins in UV-irradiated cells expressing YfgM(*p*BPA)-His_10_ were analyzed using immunoblotting with anti-His, anti-PpiD, and anti-SecG antibodies (Fig. S6). We observed six possible crosslinked products that crossreacted with anti-PpiD antibodies (YfgM derivatives with *p*BPA at Tyr-86, Val-126, Val-136, Trp-156, Trp-181, or Met-196) and three possible crosslinked products that crossreacted with anti-SecG antibodies (YfgM derivatives with *p*BPA at Asn-6, Val-11, or Glu-66). Although some of these products were not clearly detected with anti-His antibodies, all were successfully purified using nickel–nitrilotriacetic acid agarose (Fig. 3, *A* and *B*), confirming that they were indeed YfgMxPpiD or YfgMxSecG crosslinked products. The YfgM derivatives with *p*BPA at Trp-181 or Met-196 generated two crosslinked products with different mobility on SDS-PAGE (Fig. 3*A*). Probably, *p*BPA introduced at these positions in YfgM could be crosslinked with two different positions in PpiD. The functionality of the five PpiD-crosslinkable YfgM(*p*BPA) proteins and one SecG-crosslinkable YfgM(*p*BPA) protein was further examined using a VemP–PhoA reporter assay (Fig. S5*B*), which confirmed that all these proteins were functional. These findings suggest that the detected crosslinking likely reflects the physiological interactions of YfgM with PpiD and SecG, and that the *p*BPA sites introduced on YfgM are located in close proximity to the crosslinked partner proteins under physiological conditions. Therefore, we mapped the YfgM residues at which *p*BPA-mediated crosslinking with PpiD and SecG was detected onto the structural model of YfgM generated using the recently developed artificial intelligence system, AlphaFold2 (37, 38, 39) (Fig. 3*C*). Two of the three SecG-crosslinked sites (Asn-6 and Val-11; *blue spheres*) were located on the same side of a cytoplasmic α-helix N-terminally adjacent to the TM segment.

Next, we tried to identify YfgM-neighbor sites in PpiD and SecG using an *in vivo* photocrosslinking approach. Since no high-resolution structure of PpiD is currently available, we used the model PpiD–YfgM complex structure generated using AlphaFold2 (Figs. 3*D* and S7). A local distance difference test of the predicted heterodimeric structure (Fig. S7*A*, *right panel*) revealed that the model was highly accurate except for the cytoplasmic and TM regions of these proteins. According to the model PpiD–YfgM structure, the periplasmic region of PpiD had an elongated ring shape and was folded into four distinctive structural domains (Fig. S7, *B* and *C*). The N- and C-terminal parts of the PpiD periplasmic region (Ala-38 to Ala-186 and Glu-580 to Gln-622; Fig. S7*C*, in *magenta*) formed an NC domain that is structurally similar to the domains present in other PPIases (SurA, trigger factor, and PrsA) (40, 41, 42). In this model, the NC domain of PpiD interacted closely with the TPR domain of YfgM.

To verify this model, we introduced *p*BPA into three PpiD-His_10_ sites that were close to YfgM in the model (Phe-174, Ile-599, and Val-600; *green spheres* in Fig. 3*D*) and performed photocrosslinking experiments (Fig. 3*E*). We detected possible crosslinked products produced in a UV irradiation–dependent manner using anti-PpiD antibodies, finding that these products were not generated in the *yfgM* deletion strain. Mass spectrometry (MS) analysis of the purified possible crosslinked products of PpiD(F174*p*BPA)-His_10_ and PpiD(I599*p*BPA)-His_10_ showed that they contained YfgM peptides with a comparable abundance to PpiD peptides, confirming that they were indeed PpiD–YfgM crosslinked products (Fig. S8*A*). VemP–PhoA reporter assays demonstrated that these PpiD(*p*BPA) derivatives were almost fully functional (Fig. S5*C*), suggesting that the TPR domain of YfgM interacts with the NC domain of PpiD *in vivo*, as predicted by AlphaFold2. We also searched for a YfgM-binding site in SecG using the photocrosslinking approach. *p*BPA scanning targeting all regions of SecG revealed that a possible crosslinked product with YfgM was generated when *p*BPA was introduced into the Met-52 residue located in the cytoplasmic loop of SecG (Fig. 3*F*). YfgM crosslinking was confirmed by the disappearance of the crosslinked product in the *yfgM* deletion strain (Fig. 3*F*, *right panel*) and MS analysis of the purified crosslinked products of SecG(M52*p*BPA)-His_6_ (Fig. S8*B*). Together, these results support the notion that YfgM interacts with the Sec translocon *via* the cytoplasmic region of SecG.

### YfgM–PpiD complex formation is required for the interactions of these proteins with the Sec translocon

**Figure 4:**
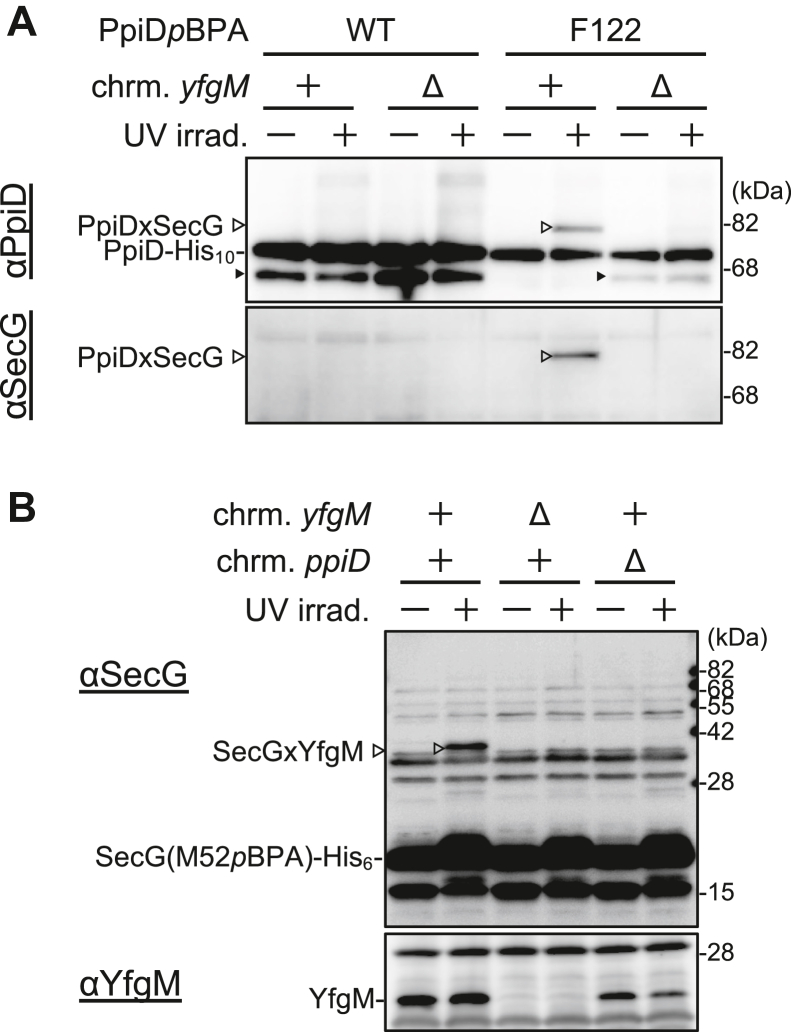
**YfgM and PpiD coexistence is crucial for interactions with SecY/E/G.***A*, effect of the *yfgM*-deletion mutation on PpiDxSecG crosslinking. RM3688 (Δ*ppiD*) or RM3690 (Δ*ppiD*, Δ*yfgM*) cells carrying both pEVOL-pBpF and pHM1021-*ppiD(F122amb)-his*_*10*_ were grown, induced, UV treated, and analyzed using SDS-PAGE and immunoblotting with the indicated antibodies, as for Figure 3*E*. *Bands* indicated with *black arrowheads* represent PpiD degradation products (PpiD′), as for Figure 3*E*. *B*, effect of *ppiD-*deletion mutation on SecGxYfgM crosslinking. NT23 (Δ*secG*), NT31 (Δ*secG*, Δ*yfgM*), or NT237 (Δ*secG*, Δ*ppiD*) cells carrying both pHM649 and pMW118-*secG(M52amb)-his*_*6*_ were grown at 30 °C in L-medium with 0.5 mM *p*BPA until midlog phase, UV-treated and analyzed using SDS-PAGE and immunoblotting with the indicated antibodies, as for Figure 3*F*.

Having demonstrated that YfgM interacts closely with PpiD *via* its TPR domain to form a stable complex (Fig. 3), that YfgM is almost as essential for VemP arrest-cancellation as PpiD (Fig. 2), and that YfgM and PpiD interact directly with the Sec translocon (Fig. 3), we further investigated PpiD–YfgM complex formation and its association with SecY/E/G using *in vivo* photocrosslinking experiments. As shown previously (25), PpiD(F122*p*BPA) generated a crosslinked product with SecG that crossreacted with both anti-PpiD and anti-SecG antibodies in the wildtype strain (Fig. 4*A*) but was not generated in the Δ*yfgM* strain (Fig. 4*A*), indicating that YfgM is crucial for PpiD(F122*p*BPA)xSecG crosslinking. Similarly, crosslinking between SecG(M52*p*BPA) and YfgM was observed in the wildtype strain (Fig. 4*B*) but not in the Δ*ppiD* strain, even though YfgM accumulated stably in the Δ*ppiD* strain, indicating that PpiD is required for YfgM(M52*p*BPA)xSecG crosslinking. Together, these results strongly suggest that YfgM–PpiD complex formation is required for each protein interact with the Sec translocon.

## Discussion

**Figure 5:**
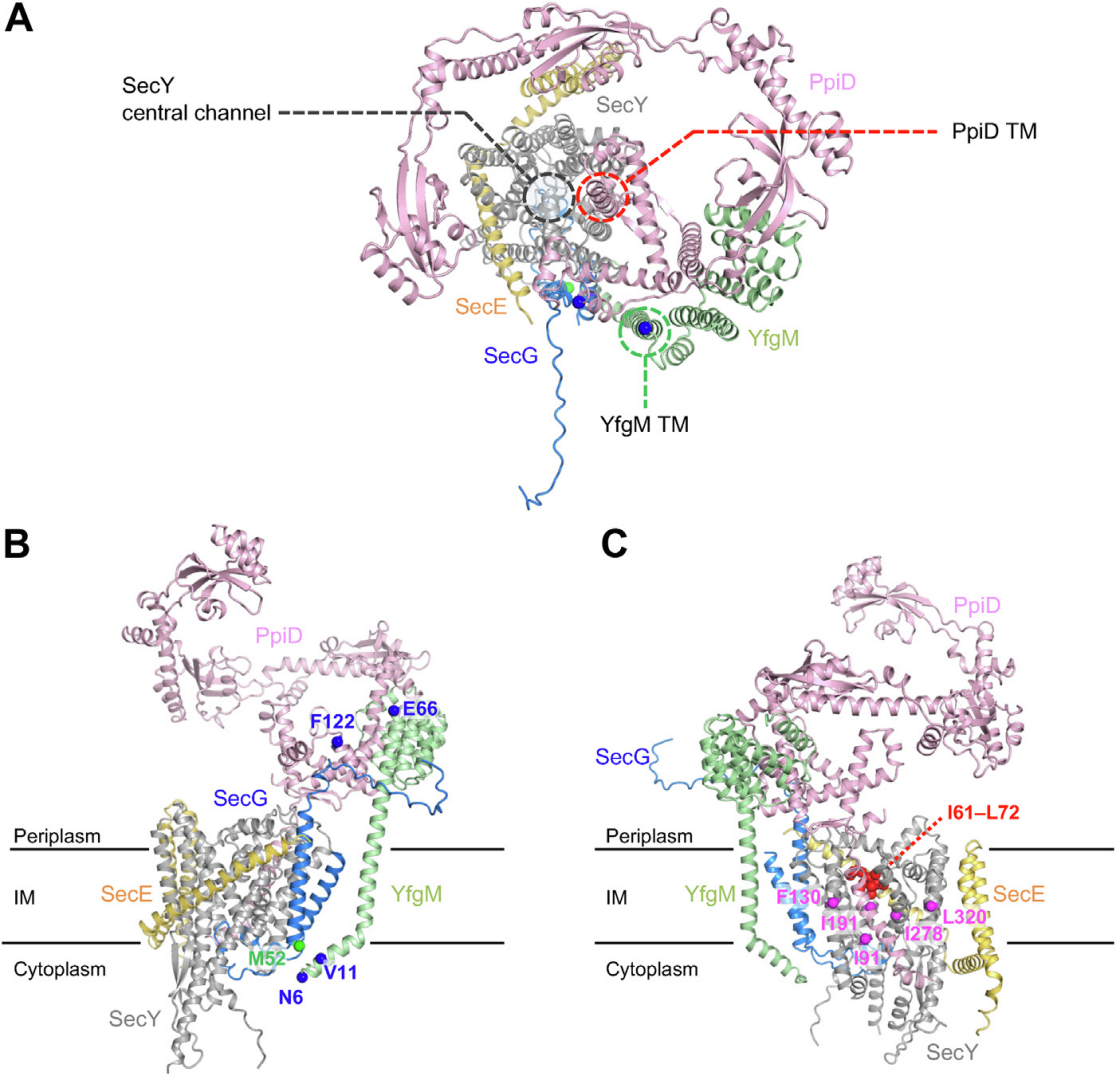
**Structural model of the SecY/E/G–PpiD/YfgM supercomplex predicted by AlphaFold2.***A*, ribbon representation of the predicted structure of an *Escherichia coli* SecY/E/G–PpiD/YfgM supercomplex viewed from the periplasmic side. The positions of the SecY central channel and the PpiD and YfgM TMs are shown as *dotted circles*. *B*, mapping of SecG-contact residues (*blue*) in YfgM and PpiD and a YfgM-contact residue (*green*) in SecG on the model structure viewed from the back side of SecY/E/G. *C*, mapping of PpiD-neighbor residues (*magenta* and *red*) in SecY on the model structure viewed from the front side of SecY/E/G. SecY, SecE, SecG, PpiD, and YfgM are indicated in *gray*, *yellow*, *blue*, *pink*, and *pale green*, respectively. TM, transmembrane.

Although it has been shown (30, 32) that YfgM forms a stable complex with PpiD, a membrane-anchored chaperone that interacts (24, 25) and presumably cooperates (25) with the Sec translocon in *E. coli*, neither the physiological functions of YfgM nor its interaction modes with PpiD and the Sec translocon have yet been clarified. In this study, we performed genetic and biochemical analyses on *yfgM* and *ppiD* mutants to explore the functions of YfgM and conducted systematic *in vivo* photocrosslinking studies to examine its interactions with PpiD and SecY/E/G. We found that (1) YfgM protects PpiD from C-terminal cleavage by unidentified protease(s), presumably by forming a functional complex (Fig. 1); (2) YfgM and PpiD play crucial roles in the TTAC of VemP (Fig. 2, *A* and *B*) (27); (3) YfgM is important for PpiD stabilization and timely VemP arrest cancellation in *V. alginolyticus*, resulting in V.SecD2 repression (Fig. 2*C*); (4) photocrosslinking analysis showed that both PpiD and YfgM directly interact with the Sec translocon and confirmed that the predicted heterodimeric structure of the PpiD–YfgM complex, in which the TPR domain of YfgM closely interacts with the NC domain of PpiD, exists *in vivo* (Fig. 3); (5) PpiD–YfgM complex formation is likely required for their proper interaction with SecY/E/G (Fig. 4). Together, these results strongly suggest that the PpiD–YfgM heterodimeric complex is a functional unit that interacts with SecY/E/G to specifically mediate the TTAC of VemP (see supporting discussion).

### PpiD–YfgM complex formation

The TPR domain, which is found in a wide variety of proteins, both in prokaryotes and eukaryotes, consists of several tandem repeats of a 34-amino acid sequence (TPR motif) that forms two antiparallel α-helices. This domain is known to mediate both the formation of stable protein complexes (43, 44) and transient protein–protein interactions, such as those that occur during protein targeting processes (44, 45, 46, 47). Here, we found that the TPR domain of YfgM, which constitutes almost the entire periplasmic region, is directly involved in the formation of the stable complex with PpiD. The structural model of PpiD–YfgM indicated a tight interaction between the TPR domain in YfgM and the NC domain in PpiD, which could stabilize heterodimer formation (Fig. 3*D*). In this model, the NC domain of PpiD was associated with the concave surface of the YfgM TPR domain, yet it remains unclear whether YfgM can directly interact with a substrate polypeptide emerging from SecY/E/G, as suggested for PpiD. Even if YfgM has such an ability, it appears that the concave surface of the TPR domain is mostly occupied with the NC domain of PpiD; however, it cannot be totally excluded that the PpiD NC domain could dissociate from the YfgM TPR domain under some conditions. It may be also possible that other parts of the TPR domain can bind a substrate (45), although our systematic photocrosslinking approach failed to detect the crosslinking of YfgM with a substrate protein, even when *p*BPA was introduced at the convex surface of the TPR domain on the opposite side of the PpiD-interacting surface. Further detailed studies are required to address the possibility of direct substrate binding by YfgM.

As mentioned previously, SurA (40), trigger factor (a ribosome-binding chaperone) (41), and PrsA (a bacterial extracellular foldase) (42) have domains that are structurally related to the NC domain of PpiD and are known to bind substrate proteins. Interestingly, the NC domain of SurA is involved in an intramolecular interaction with its inactive PPIase domain (40), whereas that of PrsA contributes toward its homodimerization, which is essential for both its substrate binding and foldase activities (42). Therefore, although the interaction modes and targets seem to differ completely between the NC domains of these chaperone proteins, they may all interact with other elements/proteins to help the individual enzymes to adopt an active conformational state. Indeed, tight interactions between the TPR domain in YfgM and the NC domain in PpiD may be crucial for the promotion of protein translocation.

It has previously been reported that the isolated periplasmic domain of PpiD, but not that of YfgM, exhibits chaperone activity *in vitro* (30), suggesting that PpiD and YfgM have separate roles in the PpiD–YfgM complex. For instance, PpiD might exhibit catalytic activity that stimulates protein folding and/or translocation by directly binding to the substrate, whereas YfgM might indirectly assist PpiD by stabilizing its functional structure and promoting its association with SecY/E/G. In the model structure of PpiD–YfgM, the periplasmic region of PpiD consisted of four distinctive structural domains connected by two interdomain loops in the following order: NC domain (most proximal to the TM segment), second domain (Q187–F223 and L477–M578), third domain (Q228–F261 and K360–K470), and parvulin (PPIase) domain (Fig. S7*C*). The second and third domains shared a similar structural fold, whereas the NC domain was composed of six α-helices in the N-terminal periplasmic region (N38–A186) and one α-helix in the C-terminal periplasmic region (E580–Q622). Since the C-terminal α-helix, which contains the Ile-599 and Val-600 residues examined in this study, appears to make extensive contacts with the TPR domain of YfgM, it might be destabilized and/or exposed in the absence of YfgM, leading to its C-terminal cleavage by unidentified protease(s), as shown in Figure 1. Each of the second, third, and parvulin domains may also play specific roles in the function of PpiD and/or the binding of a substrate protein, although the functional importance of the parvulin domains has been questioned (20). Since both the parvulin/third domains and the second/third domains were connected by two long flexible loops in the model (Fig. S7*C*), dynamic rigid-body movements of these domains could facilitate the capture of a substrate protein and interactions with partner proteins such as SecD/F during the functional cycle of PpiD. Further studies on the structure of the PpiD–YfgM complex and the interactions of PpiD with SecY/EG and SecD/F would improve our understanding of their cooperation in order to stimulate VemP arrest cancellation.

### Interaction of the PpiD–YfgM complex with SecY/E/G

The results of our *in vivo* photocrosslinking experiments in the mutant strains lacking either the *yfgM* gene or the *ppiD* gene (Fig. 4) strongly suggested that PpiD–YfgM complex formation is essential for PpiD(F122*p*BPA)–SecG and SecG(M52*p*BPA)–YfgM crosslinking. In other words, the uncomplexed PpiD and YfgM molecules were unable to stably interact with SecG, despite significant PpiD and YfgM accumulation in the Δ*yfgM* and Δ*ppiD* strains, respectively. In the PpiD–YfgM structural model, Glu-66 in the first TPR motif of YfgM and Phe-122 in the N-terminal region of PpiD, both of which were identified as SecG contact sites (Figs. 3*C* and 4) (25), were closely localized and their side chains faced in the same direction (Fig. 3*D*); therefore, it is conceivable that PpiD and YfgM in the PpiD–YfgM complex interact with the SecG subunit of the translocon in a concerted manner. The “complex” between the PpiD NC domain and the YfgM TPR domain could form a scaffold to facilitate this interaction. Since both PpiD and YfgM play critical roles in VemP maturation, we propose that the PpiD–YfgM complex is a functional unit whose formation is a prerequisite for their proper interaction with SecY/E/G in *E. coli* and *V. alginolyticus*.

When we constructed a structural model of the SecY/E/G–PpiD/YfgM supercomplex using AlphaFold2 (Fig. 5*A*), local distance difference test of the predicted structure (Fig. S9) indicated that the membrane-embedded core structure of SecY/E/G was reliable. According to this model, the TM segments of YfgM and PpiD were adjacent to SecG and the lateral gate of SecY, respectively, whereas the NC domain of PpiD was positioned just above the central channel of the Sec translocon and thus may capture emerging substrates. Our predicted structure of SecY/E/G–PpiD/YfgM is consistent with the results of this (Fig. 5*B*) and previous (Fig. 5*C*) (24, 25) *in vivo* crosslinking studies. For instance, Met-52 in SecG was in close proximity with the cytoplasmic region of YfgM in the model (Fig. 5*B*), whereas Phe-122 in PpiD and both Asn-6 and Val-11 in YfgM were close to the periplasmic region and cytoplasmic loops of SecG, respectively (Fig. 5*B*). In addition, almost all the residues identified as PpiD neighbors in the TM regions in SecY (24) were in close proximity to PpiD (*magenta* in Figs. 5*C* and S10). Although many PpiD-neighbor residues identified in the plug helix in SecY (48) looked distant from PpiD (*red* in Figs. 5*C* and S10), the findings that the plug helix is mobile and dissociates from the pore ring upon protein translocation (49) could explain this apparent discrepancy (see supporting discussion). Consequently, the predicted model could represent the structure of the SecY/E/G–PpiD/YfgM supercomplex under physiological conditions in some living cells.

PpiD is also known to contact the periplasmic domain of SecD and cooperate with the SecD/F complex during the later stages of protein translocation (28). The PpiD–YfgM complex could therefore interact with both SecY/E/G and SecD/F during protein translocation; however, it remains unclear how SecD/F physically interacts with SecY/E/G. To avoid possible steric hindrance between the large periplasmic domains of the PpiD–YfgM and SecD/F complexes, they should interact with SecY/E/G at sufficiently distant positions. Therefore, the determination of the detailed interaction modes among SecY/E/G, SecD/F, and PpiD–YfgM could improve our understanding of how a substrate polypeptide is transported from the Sec translocon to periplasmic chaperones.

### Conservation of the *yfgM* and *ppiD* genes among bacteria

Bioinformatic analyses using STRING, which enables functional protein association networks to be searched, showed that almost all bacteria carry the *ppiD* gene (Fig. S11), suggesting that PpiD plays an important role in the maturation of some exported proteins. However, only *alpha-*, *beta-*, and *gamma-proteobacteria* among gram-negative bacteria contain both the *ppiD* and *yfgM* genes, and many other bacteria lack the *yfgM* gene, including multiple gram-positive (*Firmicutes* and *Actinobacteria*) and gram-negative (*delta-proteobacteria*, *epsilon-proteobacteria*, and *Bacterioidetes*) bacteria (Fig. S11). Since a functional PpiD–YfgM complex may only exist in a limited number of bacteria, it is unclear how PpiD can function normally in bacteria that lack *yfgM.* These bacteria may possess an unidentified gene that encodes a functional counterpart of YfgM to form a complex with and assist PpiD*.* Alternatively, PpiD could stably interact with the SecY/E/G translocon alone in these bacteria to enhance protein translocation without YfgM or its functional counterpart. Studying the bacteria that lack *yfgM* will help to address this question. Based on our findings, we believe that, at least in *gamma-proteobacteria* including *E. coli* and *V. alginolyticus*, the PpiD–YfgM heterodimer acts as the functional unit to facilitate protein translocation together with SecY/E/G and SecD/F.

## Conclusion and perspective

In this study, we have demonstrated that the PpiD–YfgM heterodimer acts as a functional unit that stimulates translocation of VemP through its proper interactions with the Sec translocon and presented a structural model of the PpiD–YfgM complex in a living cell based on the experimental results. Furthermore, using AlphaFold2, we propose an artificial intelligence–based structural model of the supercomplex composed of SecY/E/G and PpiD–YfgM, which is consistent with the biochemical results of this and the previous studies and thus seems rather reliable. In the model, the ring-like–shaped large periplasmic region of PpiD is situated just above the central pore of the Sec translocon, suggesting that the periplasmic domain of PpiD contributes to functional interaction of PpiD–YfgM with a translocating substrate protein, SecD, and/or other periplasmic chaperones. A plausible working hypothesis for the PpiD–SecD-mediated arrest cancellation of VemP is that a part of the periplasmic domain of PpiD directly captures the arrested VemP and transfers it to other factors including SecD and/or other periplasmic chaperons, since it was reported that an N-terminal region of VemP physically contacts with PpiD at its arrested state (28). The next important challenge is to verify the direct interactions between the periplasmic domain of PpiD and each of these factors including VemP in living cells and reveal their molecular detail. Such information would be essential to elucidate the roles of these factors in stimulation of PpiD-dependent protein translocation. To achieve this, an *in vivo* photocrosslinking combined with AlphaFold2 prediction would be a promising approach, which was demonstrated by the identification of YfgM-contact residues in PpiD by the PpiD–YfgM model structure–instructed photocrosslinking experiments, which was much more effective as compared with the nonbiased and near exhaustive crosslinking analyses targeted to YfgM. For example, AlphaFold2-generated structural models of both SecY/E/G–PpiD/YfgM–SecD/F supercomplex and SecY/E/G–PpiD/YfgM/periplasmic chaperone supercomplex would be useful to choose target residues in PpiD and the putative partner factors, and to analyze *in vivo* interactions between these proteins. Identification of contact sites by the aforementioned procedure could enable stabilization of the complexes by a disulfide bond formation at the contact sites in order to determine their high-resolution structures by X-ray crystallographic and/or cryo-EM analyses. A similar *in vivo* photocrosslinking procedure combined with AlphaFold2 prediction would generally be useful to analyze *in vivo* interactions between proteins whose high-resolution structures are not available and provide clues for further detailed structural analysis.

## Experimental procedures

### Bacteria strains, plasmids, and primers

Bacteria strains, plasmids, and primers used in this study are listed in Tables S1, S2, and S3, respectively. Construction of the individual mutant strains and plasmids are described in the Supporting Experimental Procedures.

### Media

*E. coli* cells were grown in L rich medium (10 g/l Bacto tryptone, 5 g/l Bacto yeast extract, and 5 g/l NaCl; pH adjusted to 7.2 by using NaOH), LB-rich medium (Nacalai Tesque) and M9 synthetic medium (without CaCl_2_) (50) supplemented with all amino acids (except Met and Cys; final concentration of 20 μg/ml each), 0.4% glycerol, 0.2% maltose, and 2 μg/ml thiamine. About 50 μg/ml ampicillin, 20 μg/ml chloramphenicol, 25 μg/ml kanamycin, 50 μg/ml Spc, and 25 μg/ml tetracycline were added as appropriate for growing plasmid-bearing cells and selecting transformants and transductants. *V. alginolyticus* cells were grown in VC-rich medium (5 g/l Bacto tryptone, 5 g/l Bacto yeast extract, 4 g/l K_2_HPO_4_, 30 g/l NaCl, and 0.2% glucose) (51). About 2.5 μg/ml chloramphenicol was added for growing plasmid-bearing cells. Bacterial growth was monitored using mini photo 518R (660 nm; TAITEC).

### Antibodies

For preparation of an antibody against YfgM, two oligopeptides (C-S^65^EGKPDSIPAAEKF^78^ and C-G^165^EALLSKGDKQGAR^178^; the numbers represent the positions of the amino acid residues of *E. coli* YfgM) were chemically synthesized. These oligopeptides were mixed, conjugated with keyhole limpet hemocyanin (a carrier protein), *via* the Cys residue attached at their N terminus, and used to raise antibodies in rabbits. Penta-His horseradish peroxidase (HRP) conjugate was purchased from QIAGEN. An anti-BamB antibody was a gift from T. Shiota (Miyazaki University) (52). Anti-VemP (27), anti-V.SecD1 (27), anti-V.SecD2 (27), anti-PpiD (25), anti-SecG (53), and anti-MBP (54) antibodies were described previously. For the detection of V.PpiD, we used the anti-*E. coli* PpiD antibodies (25) that well crossreacted with V.PpiD.

### Immunoblotting analysis

This method was used in Figure 1, Figure 2, Figure 3, Figure 4, S1, S3, and S5. Total cellular proteins were solubilized, separated by SDS-PAGE, and electrotransferred onto a polyvinylidene difluoride membrane (Merck Millipore). The membrane was first blocked with 1% or 5% skim milk in PBST (PBS [136 mM NaCl, 2.7 mM KCl, 4.3 mM Na_2_PO_4_, 1.5 mM KH_2_PO_4_] with 0.1% Tween-20) and then incubated with anti-YfgM (1/2000 dilution), anti-PpiD (1/20,000 or 1/50,000 dilution), anti-BamB (1/2000 dilution), anti-His (1/2000 dilution), anti-V.SecD1 (1/2000 dilution), or anti-V.SecD2 (1/2000 dilution) antibodies in the blocking solutions at room temperature overnight. After washing with PBST three times, the membrane was incubated with a HRP-conjugated secondary antibody) (1/5000 dilution; Goat Anti-Rabbit IgG (H + L)-HRP conjugate; Bio-Rad Laboratories, Inc) in PBST at room temperature for 1 h. Proteins were visualized with detection reagents (ECL Western Blotting Detection Reagents [GE Healthcare UK Ltd], ECL Prime Western Blotting Detection Reagents [GE Healthcare], and Chemi-Lumi One [Nacalai Tesque]), and chemiluminescence image analyzers (LAS3000 mini lumino-image analyzer [GE Healthcare], LAS4000 mini lumino-image analyzer [GE Healthcare], and FUSION Solo S [VILBER]).

### Pulse-chase analysis of *in vivo* stability of the arrested form of *VemP*

The procedure was used in Figs 2 and S3. Cells were first grown at 30 °C in M9-medium supplemented with 18 kinds of amino acids (except Met and Cys), 0.4% glycerol, 0.2% maltose, and 2 μg/ml thiamine. Cells were induced with 1 mM IPTG for 15 min and pulse-labeled with 370 kBq/ml [^35^S]Met (American Radiolabeled Chemicals, Inc) for 30 s. Chase was initiated by adding excess nonradioactive Met (final concentration of 250 μg/ml) to the culture. At appropriate time points after the addition of cold Met, total cellular proteins were immediately precipitated with 5% trichloroacetic acid, washed with acetone, and solubilized in SDS buffer (50 mM Tris–HCl [pH 8.1], 1% SDS, 1 mM EDTA). The samples were applied to immunoprecipitation with anti-VemP antibodies as described previously (28). Isolated proteins were separated by SDS-PAGE and visualized with BAS5000 phosphor imager.

### *In vivo* photocrosslinking analysis

These methods were used in Figures 3, 4, and S5.

The crosslinking experiment with the *p*BPA-containing derivatives of YfgM, PpiD, and SecG was carried out as follows.

YfgM*p*BPA: Cells of NT35 (Δ*yfgM*) carrying pEVOL-pBpF and pMW118-*yfgM(amb)-his*_*10*_ were grown at 30 °C in L medium containing 0.5 mM *p*BPA until early log phase and induced with 1 mM IPTG for 1 h.

PpiD*p*BPA: Cells of RM3688 (Δ*ppiD*) or RM3690 (Δ*ppiD*, Δ*yfgM*) carrying pEVOL-pBpF and pHM1021-*ppiD(amb)-his*_*10*_ were grown at 30 °C in L medium containing 0.5 mM *p*BPA and 0.02% arabinose until early log phase and induced with 1 mM IPTG for 1 h.

SecG*p*BPA: Cells were grown at 30 °C in L medium containing 1 mM *p*BPA until midlog phase.

The half volume of the cell cultures described above was put on a petri dish at 4 °C and UV irradiated for 10 min using B-100AP UV lamp (365 nm; UVP, LLC), at a distance of 4 cm. The other half was kept on ice as non–UV-irradiated samples. Total cellular proteins were precipitated with 5% trichloroacetic acid, washed with acetone, and suspended in SDS-sample buffer. Crosslinking products of YfgM(*p*BPA) shown in Figure 3*A* and *B* were partially purified according to the procedure described previously (26). In the case of the SecG*p*BPA derivative, the UV-treated and nontreated cells were collected by centrifugation, washed with 10 mM Tris–HCl (pH 8.1), and suspended in buffer A (50 mM Tris–HCl [pH 7.5], 1 mM EDTA, and 0.1 mM Pefabloc [Merck]). Cells were disrupted by freeze–thawing and sonication with cooling on ice. After removal of debris materials by centrifugation, membranes were precipitated by ultracentrifugation and solubilized with SDS-sample buffer.

All the samples were subjected to SDS-PAGE and immunoblotting analysis.

### Purification and MS analysis of crosslinked products

The *p*BPA-containing PpiD and SecG derivatives were purified as follows.

PpiD*p*BPA: Cells of RM3688 (Δ*ppiD*) carrying pEVOL-pBpF and either pHM1021-*ppiD(F174amb)-his*_*10*_ or pHM1021-*ppiD(I599amb)-his*_*10*_ were grown and induced as described above. Cells were suspended in 20 mM Tris–HCl (pH 8.0), treated with or without UV irradiation for 20 min using B-100AP, and disrupted by sonication at 0 °C. After precipitation of total membranes by ultracentrifugation of cell lysates at 100,000*g* for 60 min, they were solubilized with 1% SDS buffer (50 mM Tris–HCl [pH 8.0], 300 mM NaCl, and 1% SDS) and centrifuged at 10,000*g* for 5 min. Supernatants were 10-fold diluted with a buffer containing 50 mM Tris–HCl (pH 8.0) and 300 mM NaCl and incubated with TALON resin (Takara Bio) at room temperature for 2.5 h with rotation. After washing the resin with wash buffer (50 mM Tris–HCl [pH 8.0], 300 mM NaCl, and 0.1% SDS), proteins were eluted with SDS-sample buffer.

SecG*p*BPA: Cells of SA101 (Δ*secG*) carrying pHM649 and either pTWV228-*secG(M52amb)-his*_*6*_ or pTWV228-*secG-his*_*6*_ were grown at 37 °C in 1 l of M9-synthetic medium supplemented with 0.4% glucose, 5 mM CaCl_2_, and 1 mM *p*BPA until midlog phase. Cells collected by centrifugation were washed with 10 mM Tris–HCl (pH 8.1), suspended in buffer A (50 mM Tris–HCl [pH 8.1], 1 mM EDTA, 0.1 mM Pefabloc [Sigma–Aldrich]), and treated with or without UV irradiation for 30 min using B-100AP at 4 °C. Then, the cells were disrupted by French pressure cell press (SLM-Aminco) at 4 °C. After clarification by centrifugation, membranes were recovered by ultracentrifugation and suspended in buffer A. The suspension was mixed with an equal volume of 0.2 M NaOH to denature and remove peripheral membrane proteins. The alkaline-treated membranes were collected by centrifugation and solubilized with buffer A containing 1% SDS. After clarification, a portion of the supernatants was mixed with an appropriate volume of 300 mM NaCl, 0.2 mM EDTA, H_2_O, and 10% SDS to obtain a solution with final concentrations of 150 mM NaCl, 0.1 mM EDTA, and 0.5% SDS. The diluted sample was incubated with TALON resin for 2 h with mild rotation at room temperature. After washing the resin with buffer B (50 mM Tris–HCl [pH 8.0], 150 mM NaCl, and 0.5% SDS) containing 3 mM imidazole, proteins were eluted with buffer B containing 81 mM EDTA. Protein-enriched fractions were concentrated by acetone precipitation and solubilized with SDS sample buffer.

The purified crosslinked products were separated on SDS-PAGE and silver stained with Sil-Best Stain One (Nacalai Tesque). The bands were excised and digested in gel with a TPCK-trypsin (Worthington Biochemical). Then digest was analyzed by nano liquid LC–MS/MS. In the case of PpiD*p*BPA, the peptides were separated using nano ESI spray column (75 μm [ID] × 100 mm [L], NTCC analytical column C18, 3 μm; Nikkyo Technos) with a linear gradient of buffer B (100% acetonitrile and 0.1% formic acid) at a flow rate of 300 nl/min (Easy nLC; Thermo Fisher Scientific). The Q Exactive mass spectrometer (Thermo Fisher Scientific) was operated in the positive-ion mode, and the MS and MS/MS spectra were acquired in a data-dependent TOP10 method. Obtained MS raw data were processed with Proteome Discoverer 2.4 (Thermo Fisher Scientific) using MASCOT program V2.7 (Matrix Science) as a sequence database search node. For SecG*p*BPA analysis, LCQ Deca XP (Thermo Fisher Scientific) was used for tandem mass spectrometer, and the obtained MS/MS data were searched for the National Center for Biotechnology Information nr database using MASCOT program.

### PhoA assay using the VemP–PhoA reporter protein

PhoA activity of NT17 derivatives expressing the reporter protein, VemP–PhoA, was measured as follows. Cells of NT17 derivatives carrying pHM1550-*vemP-phoA* were grown in LB medium supplemented with 1 mM IPTG at 30 °C for 2.5 h. A portion of the cell cultures was collected, washed with, and resuspend in 1 M Tris–HCl (pH 8.1). Cells were lysed by treatment with CHCl_3_ (final concentration of 5%) and SDS (final concentration of 0.025%), mixed with *p*-nitrophenylphosphate (a chromogenic substrate of PhoA; Sigma, final concentration of 0.04%), and incubated for 1 h at 37 °C. The reaction was stopped by addition of excess KH_2_PO_4_. After clarification by centrifuge, absorbance at 420 and 550 nm of each culture was measured by nanodrop. PhoA activity of the cells expressing both a PpiD(*p*BPA) derivative and VemP–PhoA was measured as follows. RM3688 (Δ*ppiD*) carrying pEVOL-pBpF, pHM1550-*vemP-phoA*, and pHM1021-*ppiD(amb)*-*his*_*10*_ were grown in LB medium supplemented with 1 mM IPTG in the presence or the absence of 0.5 mM *p*BPA at 30 °C for 3 h. Cell lysis and PhoA activity measurement were conducted as described above. PhoA activity of the cells expressing both a YfgM(*p*BPA) derivative and VemP–PhoA was measured as follows. Cells of NT35 (Δ*yfgM*) carrying pEVOL-pBpF, pHM1550-*vemP-phoA*, and pTWV228-*yfgM(amb)*-*flag* were grown in L-medium supplemented with 1 mM IPTG in the presence or the absence of 0.5 mM *p*BPA at 30 °C for 3 h. A portion of the cell cultures was collected, washed with 1 M Tris–HCl (pH 8.1), and resuspended in equal volume of 1 M Tris–HCl (pH 8.1). Cells were lysed by treatment with CHCl_3_ and SDS. Then, 72 μl of the supernatant was mixed vigorously with 108 μl of 1 M Tris–HCl (pH 8.1) and 20 μl of 0.4% *p*-nitrophenylphosphate in a clear 96-well plate and incubated for 40 min at room temperature. During this incubation, absorbance at 420 and 550 nm was measured at every 2 min for each well using the Viento Nano microplate reader (BioTek Instruments, Inc).

PhoA activity in arbitrary units was calculated according to the equation: PhoA activity (arbitrary units) = (absorbance at 420 nm - 1.75 × absorbance at 550 nm)/(incubation time [min] × [absorbance at 600 nm of the bacterial culture at the time of harvest]).

### Structural prediction with AlphaFold2

The structural model of *E. coli* YfgM was obtained from AlphaFold2 Protein Structure Database (https://alphafold.ebi.ac.uk/). The structural models of *E. coli* PpiD–YfgM heterodimer and PpiD–YfgM–SecY/E/G supercomplex were predicted by ColabFold (55) installed on a local workstation using amino acid sequences of *E. coli* PpiD, YfgM, SecY, SecE, and SecG. From the five structural models of each complex that were generated by the prediction, we only showed a single model because they were similar to each other. The coordinates of *E. coli* PpiD–YfgM heterodimer and PpiD–YfgM–SecY/E/G supercomplex are provided as a Protein Data Bank format in Files S1 and S2, respectively.

## Data availability

All data described are contained within the article.

## Supporting information

This article contains supporting information including references (56, 57, 58, 59, 60, 61, 62, 63, 64, 65, 66, 67, 68).

## Conflict of interest

The authors declare that they have no conflicts of interest with the contents of this article.
