# Supplementary material for: Inner membrane YfgM–PpiD heterodimer acts as a functional unit that associates with the SecY/E/G translocon and promotes protein translocation
Source: J Biol Chem. 2022 Oct 7;298(11):102572. doi: 10.1016/j.jbc.2022.102572 (PMC9643414; doi:10.1016/j.jbc.2022.102572)
Supplement: Supporting information [file mmc1.pdf]

Supporting information

Inner membrane YfgM–PpiD heterodimer acts as a functional unit that associates with the SecY/E/G translocon and promotes protein translocation

Ryoji Miyazaki[1], Mengting Ai[2,#], Natsuko Tanaka[2,#], Takehiro Suzuki[3], Naoshi Dhomae[3], Tomoya Tsukazaki[1], Yoshinori Akiyama[2], Hiroyuki Mori[2,*]

Material included:

Supporting discussion (page S-2)

Supporting experimental procedures (pages S-3 and S-4)

Table S1 (page S-5)

Table S2 (pages S-6 – S-8)

Table S3 (page S-9)

Figure S1 (page S-10)

Figure S2 (page S-11)

Figure S3 (page S-12)

Figure S4 (page S-13)

Figure S5 (page S-14)

Figure S6 (page S-15)

Figure S7 (page S-16)

Figure S8 (page S-17)

Figure S9 (page S-18)

Figure S10 (page S-19)

Figure S11 (page S-20)

File S1 (PDB format)

File S2 (PDB format)

Supporting Discussion

Different requirement of the PpiD/YfgM complex for the TTAC of monitoring substrates

Requirement of the PpiD/YfgM complex for the TTAC of VemP is consistent with the previous observations that the TTAC of VemP specifically occurs at a late step of its translocation on the SecY/E/G complex after processing of its signal sequence (36), and that PpiD cooperatively functions with SecD to mediate the TTAC of VemP (28). On the other hand, Jauss *et al.* reported that the TTAC of the LepB-SecM chimeric protein normally undergoes even in the absence of both of YfgM and PpiD (48). This results could also match the finding that the TTAC of SecM, another monitoring substrate, occurs at an early step of its translocation before its signal sequence cleavage (36), where the PpiD/YfgM complex is presumably unable to function. The difference of these monitoring substrates in the timing of the TTAC could have been evolved to be suitable for their physiological functions. Indeed, to delay the timing of the TTAC, VemP would have acquired the Arg-85 residue essential for the SecD/PpiD-dependent arrest cancelation (28), although the detail molecular mechanism remains unsolved. As a result, VemP could specifically monitor the activity of SecD/F that plays a role in a late step of translocation (27) and SecM could monitor the activity of SecA, the essential motor ATPase acting at an initial step of translocation (56).

Reliability of the AI-predicted model structure

The previous *in vivo* photo-crosslinking study showed that several amino acid residues in the TM segments of SecY contacted with PpiD (24). Mapping of these residues on the AI-predicted PpiD/YfgM-SecY/E/G super-complex structure (magenta in **Fig. S10**) reveals that almost all of the identified PpiD-neighbor residues, except for the Leu-320 in SecY, are in close proximity to PpiD, suggesting that the model structure is highly reliable. Since the interactions between SecY and PpiD should be not static but dynamic, it is supposed that the interaction between the Leu-320 of SecY and PpiD could occur at a different conformational state of the complex. On the other hand, another *in vivo* photo-crosslinking study indicated that many residues in the plug helix in SecY also interacted with PpiD (48). The fact that most of these residues are mapped to the positions distant from PpiD on the model structure (red in **Fig. S10**) might apparently undermine the credibility of the model structure. However, because it was reported that the plug helix has a highly dynamic nature (49) and dissociates from the pore-ring to generate a vertical pore, when protein translocation starts (1, 3), the positions of the plug helix in the SecY/E/G translocon should be different between in a resting state (crystal structure) and in functional states. During protein translocation, the plug helix would occupy several different positions in SecY/E/G. Under some conformational states, it could be in close proximity to PpiD. Thus, it is supposed that the AI-predicted model structure of PpiD/YfgM-SecY/E/G does not necessarily conflict with the crosslinking results, because the model structure would represent a resting state in which such large conformational changes of the plug helix have not been taken into consideration.

Supporting Experimental Procedures

Construction of Mutant Strains

NT17 (MC4100, Δ*ompT*) was constructed by transducing Δ*opmT*::*kan* from JW0554 (57) to MC4100 (58) and then removing the *kan* cassette with pCP20 (59). NT23 (NT17, Δ*secG*) was constructed by transducing Δ*secG*::*kan* from JW3142 (57) to NT17 and then removing the *kan* cassette with pCP20. NT31 (NT17, Δ*secG* Δ*yfgM*) and NT35 (NT17, Δ*yfgM*) was constructed by transducing Δ*yfgM*::*kan* from JW2497 (57) to NT23 and NT17, respectively, and then removing the *kan* cassette with pCP20. NT237 (NT17, Δ*secG* Δ*ppiD*) was constructed by transducing Δ*ppiD*::*kan* from JW0431 (57) to NT23 and then removing the *kan* cassette with pCP20. RM3688 (NT17, Δ*ppiD*::*kan*) and RM3690 (NT35, Δ*ppiD*::*kan*) were constructed by transducing Δ*ppiD*::*kan* from JW0431 (57) to NT17 and NT35, respectively. HM5894(NT17, *ppiD-his*$_{10}$::*kan*) and HM5895 (NT17, Δ*yfgM* *ppiD-his*$_{10}$::*kan*) were constructed by transducing *ppiD-his*$_{10}$::*kan* from RM3032 (28) to NT17 and NT35, respectively.

NT134 (NT17, *yfgM(TM f.s.) zff-208*::Tn*10*) was constructed as follows. HM3663 (*polA1 zff-208*::Tn*10*) was transformed with a plasmid pHM764 (pK19mobsacB-*yfgM(TM f.s.)*) (see below) and a kanamycin (12.5 μg/mL)-resistant strain, in which pHM764 had been integrated into the chromosome by single crossover homologous recombination, was obtained. Then, the strain in which the plasmid sequence was excised was selected by growing the pHM764-integrated strain on P medium (20 g/L polypeptone, 5 g/L NaCl; pH adjusted to 7.2 by using NaOH*)* with 5% sucrose at 37 °C. One of the segregants that had the *yfgM(TM f.s.)* mutations on the chromosome was used as a donor in P1 transduction to transfer *yfgM(TM f.s.)* to NT17, yielding NT134. NT135 (NT17, *yfgM*$^+$ *zff-208*::Tn*10*) was obtained as an isogenic *yfgM*$^+$strain of NT134.

Vibrio mutant strain RMV6 was constructed using a "suicide vector", pSW7848, carrying the toxin-encoding *ccdB* gene under the arabinose promoter control (27). 150 μL of an overnight culture of *E. coli* β3914 cells harboring pRM742 (pSW7848-Δ*Va.yfgM*) was mixed with 50 μL of an overnight culture of *V. alginolyticus* VIO5 strain. Cells were harvested and resuspended in 100 μL of the VC medium and 2.5 μL of the suspension was spotted on the VC agar medium containing 300 μM 2,6-diaminopimelic acid (DAP) and incubated at 30 °C for 6 h. Then the grown cells were streaked on a VC plate containing 2.5 μg/mL chloramphenicol but not containing DAP to select *Vibrio* cells in which the plasmid had been integrated on the chromosome. Subsequently, the chloramphenicol-resistant bacteria were grown on VC agar plates containing 0.2% arabinose instead of glucose to counter-select the plasmid-integrated *Vibrio* cells. After confirmation of chloramphenicol-sensitivity and arabinose resistance of the obtained cells, presence of the introduced mutations was confirmed by colony PCR.

Plasmids Construction

pNT16 (pMW118-*yfgM-his*$_6$) was constructed as follows. A *yfgM-his*$_6$ fragment was PCR-amplified from genomic DNA prepared from MC4100 using the primers, yfgM-f and yfgM-his$_6$-r, digested with KpnI and HindIII, and cloned into the same sites of pMW118. pRM693 (pMW118-*yfgM-his*$_{10}$) was constructed from pNT16 by site-directed mutagenesis using a pair of appropriate primers. pMW118-*yfgM(amb)-his*$_{10}$ plasmids were constructed from pRM693 by site-directed mutagenesis using a pair of appropriate primers. pNT9 (pTWV228-*yfgM-flag*) was constructed as follows. A *yfgM-flag* fragment was PCR-amplified from the genomic DNA prepared from MC4100 using the primers, yfgM-f and yfgM-flag-r, digested with KpnI and SphI, and cloned into the same sites of pTWV228. pTWV228-*yfgM(amb)-flag* plasmids were constructed from pNT9 by site-directed mutagenesis using a pair of appropriate primers.

pRM899 (pHM1021-*ppiD-his*$_{10}$) was constructed as follows. A *ppiD-his*$_{10}$ fragment was PCR-amplified from MC4100 genome using ppiD-f and ppiD-his$_{10}$-r primers, digested with NcoI and BamHI and cloned into the same sites of pTV118N (Takara Bio). The *ppiD-his*$_{10}$ fragment of the resultant plasmid was then sub-cloned into the same sites of

pHM1021(27). pHM1021-*ppiD(amb)-his*$_{10}$ plasmids were constructed from pRM899 by site-directed mutagenesis using a pair of appropriate primers.

pGK52 (pTWV228-*secG(M52amber)*) was constructed from pTWV228-*secG* (lab stock) by site-directed mutagenesis using a pair of appropriate primers. pNT1 (pTWV228-*secG(M52amber)-his*$_6$) was constructed as follows. A *secG(M52amber)-his*$_6$ fragment was PCR-amplified from pGK52 using the primers, RV-N and secG-his$_6$-r, digested with SacI and HindIII, and cloned into the same sites of pTWV228.

pHM1258 (pHM1021-*vemP-phoA*) was constructed as follows. A NcoI-MfeI fragment prepared from pTS48 (pTWV228-*vemP-f3m*) (28) was cloned into the same sites of pHM1144 (pHM1021-Δ*ss-vemP-lacZ*) (36) yielding pEI214. Separately, a DNA fragment encoding the mature region of PhoA (22-471) was PCR-amplified from the genomic DNA prepared from *E. coli* AD16 (60) using the primers, PhoA-BamHI-F2 and PhoA-HindIII-R, digested with BamHI and HindIII and cloned into the same sites of pEI214 to obtain pEI234. A NcoI site in the *phoA* region of pEI234 was mutated without causing an amino acid substitution by site-directed mutagenesis using a pair of appropriate primers to obtain pHM1258. pHM1550 (pMW118 (Spc)) was constructed as follows. A 2.1 Kbp HindIII fragment containing a *spc*^{R} gene was prepared from pHM45Ω (61). Separately, a vector fragment without a *bla* gene was PCR-amplified from pMW118 using the pair of primers, pMW-Spc-for and pMW-Spc-rev. The *spc*^{R} gene fragment and the pMW118-derived fragment were connected using In-Fusion® HD cloning kit (Clontech) to obtain pHM1548. Then, a unique NcoI site was introduced at the start codon of *lacZα* of pHM1548 by site-directed mutagenesis using a pair of appropriate primers to generate pHM1550. pHM1552 (pHM1550-*vemP-phoA*) was constructed as follows. A NcoI-HindIII fragment containing the *vemP-phoA* gene was prepared from pHM1258 and cloned into the same sites of pHM1550 to obtain pHM1552.

pHM764 (pK19mobsacB-*hisS-yfgM(TM f.s.)-bamB*) used for construction of NT134 (NT17, *yfgM(TM f.s.) zff-208*::Tn*10*) was constructed as follows. First, an about 3 kbp fragment containing *hisS-yfgM-bamB* genes was PCR-amplified from the genomic DNA prepared from MC4100 using hisS-f and bamB-r primers, digested with SalI and SphI, and cloned into the same sites of pK18mobsacB to obtain pHM763. Next, the frame shift mutations in *yfgM* shown in Fig. 1*D* were introduced into the pHM763 by two successive site-directed mutagenesis reactions using the pair of primers, G ins-f and its complementary oligonucleotide and another pair of primers, C del-f and its complementary oligonucleotide to obtain pHM764.

pRM742 used for construction of RMV6 (VIO5, Δ*yfgM*) was constructed as follow. First, a DNA fragment containing the *V.yfgM* gene with (~1 kb) upstream and downstream sequences was amplified from the genome of VIO5 using Va-yfgM-f and Va-yfgM-r primers, and ligated with the BamHI- and SalI-digested pUC118 fragment using In-Fusion HD cloning Kit to generate pRM735. Next, a DNA fragment of the entire vector sequence with the *V.yfgM*-upstream sequence at one end and the *V.yfgM*-downstream sequence at the other end was amplified from pRM735 by PCR using del-Va-yfgM-f and del-Va-yfgM-r primers, and self-ligated using In-Fusion HD cloning Kit to produce pRM738. Finally, ~2 kbp DNA fragment containing *V.ppiD*-upstream and -downstream regions was amplified from a pRM738 using pSW-yfgM-f and pSW-yfgM-r primers, and ligated with the NaeI-digested pSW7848 using In-FusionR HD cloning Kit to yield pRM742.

Table S1. Strains used in this study.

(A) *E. coli* strains

Strain	Genotype	Reference
MC4100	F^- *araD139* Δ*(argF-lac)U169 rpsL150 relA1 flbB5301 deoC1 ptsF25 rbsR*	(58)
NT17	MC4100, Δ*ompT*	This study
NT35	NT17, Δ*yfgM*	This study
RM3688	NT17, Δ*ppiD*::*kan*	This study
RM3690	NT17, Δ*yfgM* Δ*ppiD*::*kan*	This study
NT135	NT17, $yfgM^+$ *zff-208*::Tn*10*	This study
NT134	NT17, *yfgM(TM f.s.) zff-208*::Tn*10*	This study
HM5894	NT17, $ppiD\text{-}his_{10}$::*kan*	This study
HM5895	NT17, Δ*yfgM* $ppiD\text{-}his_{10}$::*kan*	This study
NT23	NT17, Δ*secG*	This study
NT31	NT17, Δ*secG* Δ*yfgM*	This study
NT237	NT17, Δ*secG* Δ*ppiD*	This study
AD202	MC4100, *ompT*::*kan*	(62)
SA101	AD202, *secG*::*kan zha-203*::Tn*10*	(63)

(B) *V. alginolyticus* strains

Strain	Genotype	Reference
VIO5	138-2 Rif^R Pof^+ Laf^-	(64)
NR23	VIO5, Δ*V.secD1/F1*::*kan*	(27)
HM3740	VIO5, Δ*V.secD2/F2*::*kan*	(27)
RMV2	VIO5, Δ*V.ppiD*	(28)
RMV6	VIO5, Δ*V.yfgM*	This study

Table S2. Plasmids used in this study.

Plasmid	Vector	Encoded gene and description	Reference or source
pEVOL-pBpF		p15A-derivative encoding an evolved *M. jannaschii* aminoacyl-tRNA synthetase/ suppressor tRNA pair for incorporation of *p*BPA; Cm^R	(65)
pHM649		p15A-derivative encoding an evolved *M. jannaschii* aminoacyl-tRNA synthetase/ suppressor tRNA pair for incorporation of *p*BPA; Cm^R	(66)
pMW118		Expression vector; P_{lac}, Amp^R	Takara Bio
pRM693	pMW118	*yfgM-his*$_{10}$	This study
pRM694	pMW118	*yfgM(N6amb)-his*$_{10}$	This study
pRM695	pMW118	*yfgM(V11amb)-his*$_{10}$	This study
pRM696	pMW118	*yfgM(R16amb)-his*$_{10}$	This study
pRM697	pMW118	*yfgM(N21amb)-his*$_{10}$	This study
pRM698	pMW118	*yfgM(A26amb)-his*$_{10}$	This study
pRM699	pMW118	*yfgM(L31amb)-his*$_{10}$	This study
pRM700	pMW118	*yfgM(L36amb)-his*$_{10}$	This study
pRM701	pMW118	*yfgM(Y41amb)-his*$_{10}$	This study
pRM702	pMW118	*yfgM(Q46amb)-his*$_{10}$	This study
pRM703	pMW118	*yfgM(R51amb)-his*$_{10}$	This study
pRM704	pMW118	*yfgM(A56amb)-his*$_{10}$	This study
pRM705	pMW118	*yfgM(V61amb)-his*$_{10}$	This study
pRM706	pMW118	*yfgM(E66amb)-his*$_{10}$	This study
pRM707	pMW118	*yfgM(S71amb)-his*$_{10}$	This study
pRM708	pMW118	*yfgM(E76amb)-his*$_{10}$	This study
pRM709	pMW118	*yfgM(E81amb)-his*$_{10}$	This study
pRM710	pMW118	*yfgM(Y86amb)-his*$_{10}$	This study
pRM711	pMW118	*yfgM(S91amb)-his*$_{10}$	This study
pRM712	pMW118	*yfgM(Q96amb)-his*$_{10}$	This study
pRM713	pMW118	*yfgM(K101amb)-his*$_{10}$	This study
pRM714	pMW118	*yfgM(K106amb)-his*$_{10}$	This study
pRM715	pMW118	*yfgM(L111amb)-his*$_{10}$	This study
pRM716	pMW118	*yfgM(A116amb)-his*$_{10}$	This study
pRM717	pMW118	*yfgM(E121amb)-his*$_{10}$	This study
pRM718	pMW118	*yfgM(V126amb)-his*$_{10}$	This study
pRM719	pMW118	*yfgM(L131amb)-his*$_{10}$	This study
pRM720	pMW118	*yfgM(V136amb)-his*$_{10}$	This study

pRM721	pMW118	*yfgM(A141amb)-his*$_{10}$	This study
pRM722	pMW118	*yfgM(K146amb)-his*$_{10}$	This study
pRM723	pMW118	*yfgM(I151amb)-his*$_{10}$	This study
pRM724	pMW118	*yfgM(W156amb)-his*$_{10}$	This study
pRM725	pMW118	*yfgM(A161amb)-his*$_{10}$	This study
pRM726	pMW118	*yfgM(E166amb)-his*$_{10}$	This study
pRM727	pMW118	*yfgM(K171amb)-his*$_{10}$	This study
pRM728	pMW118	*yfgM(G176amb)-his*$_{10}$	This study
pRM729	pMW118	*yfgM(W181amb)-his*$_{10}$	This study
pRM730	pMW118	*yfgM(K186amb)-his*$_{10}$	This study
pRM731	pMW118	*yfgM(P191amb)-his*$_{10}$	This study
pRM732	pMW118	*yfgM(M196amb)-his*$_{10}$	This study
pRM733	pMW118	*yfgM(I201amb)-his*$_{10}$	This study
pRM734	pMW118	*yfgM(I206amb)-his*$_{10}$	This study
pHM1021		Expression vector; P_{lac}, Amp^R	(27)
pTS48	pHM1021	*vemP-3xflag-myc*	(28)
pRM899	pHM1021	*ppiD-his*$_{10}$	This study
pRM978	pHM1021	*ppiD(F122amb)-his*$_{10}$	This study
pRM947	pHM1021	*ppiD(F174amb)-his*$_{10}$	This study
pRM948	pHM1021	*ppiD(I599amb)-his*$_{10}$	This study
pRM949	pHM1021	*ppiD(V600amb)-his*$_{10}$	This study
pTWV228		Expression vector; P_{lac}, Amp^R	Takara Bio
pNT9	pTWV228	*yfgM-flag*	This study
pAI20	pTWV228	*yfgM(V11amb)-flag*	This study
pAI13	pTWV228	*yfgM(L36amb)-flag*	This study
pAI14	pTWV228	*yfgM(R51amb)-flag*	This study
pAI15	pTWV228	*yfgM(Y86amb)-flag*	This study
pAI16	pTWV228	*yfgM(Q96amb)-flag*	This study
pAI21	pTWV228	*yfgM(L111amb)-flag*	This study
pAI17	pTWV228	*yfgM(V126amb)-flag*	This study
pAI18	pTWV228	*yfgM(W156amb)-flag*	This study
pAI22	pTWV228	*yfgM(W181amb)-flag*	This study
pAI19	pTWV228	*yfgM(M196amb)-flag*	This study
pNT1	pTWV228	*secG(M52amb)-his*$_6$	This study
pHM1550		Expression vector; P_{lac}, Spc^R	This study
pHM1552	pHM1550	*vemP-phoA*	This study
pK18mobsacB		vector for chromosomal gene replacement carrying *sacB*; Km^R	(67)
pHM764	pK19mobsacB	*hisS yfgM (TM f.s.) bamB*	This study

pUC118		Expression vector; P_{lac}, Amp^R	Takara Bio
pRM735	pUC118	*Va.yfgM* +up- & down-stream 1kb	This study
pRM738	pUC118	Δ*Va.yfgM* +up- & down-stream 1kb	This study
pSW7848		R6K γ-ori-based suicide vector, P_{araBAD}-*ccdB*, Cm^R	(68)
pRM742	pSW7848	Δ*Va.yfgM* +up- & down-stream 1kb	This study

Table S3. Primers used in this study.

Name	Sequence (5' to 3')
yfgM-f	AATAATGGTACCGGGTTAAGGAAGGAGAAGGAC
yfgM-his$_6$-r	AGTGCCAAGCTCAATGGTGATGGTGATGGTGGATGGACAAATTATTAATTTT
yfgM-flag-r	ATAATAGCATGCTCATTTGTCGTCGTCGTCTTTATAGTCGATGGACAAATTATTAATTTT
ppiD-f	GCGCCCATGGCGATGGACAGCTTACGCACG
ppiD-his$_{10}$-r	GCGCGGATCCTTAATGATGATGATGATGATGATGATGATGATGTTGCTGTTCCAGCGC
RV-N	CAGGAAACAGCTATGAC
secG-his$_6$-r	AGTGCCAAGCTTAGTGGTGATGGTGATGGTGGTTCGGGATATCGCTGGTCG
PhoA-BamHI-F2	AAAATAGGATCCGCGGACACCAGAAATGCC
PhoA-HindIII-R	AAATAAGCTTTTATTTCAGCCCCAGAGCGG
pMW-Spc-for	TACAAGCATAAAGCTGCATTGGTAACTGTCAGACC
pMW-Spc-rev	TACAAGCATAAAGCTAGGGCGACACGGAAATGTTG
hisS-f	CCCGCATGCCTCCCGAAACATTGAGGG
bamB-r	GCCATTATACAGCACCGG
G-ins-f	GCTGAAAATGGCAAGAGCACTGGCTGTTGG
G-ins-r	CCAACAGCCAGTGCTCTTGCCATTTTCAGC
C-del-f	CTGGCGCTACTGGAAAGCCATCAGGTTG
C-del-r	CAACCTGATGGCTTTCCAGTAGCGCCAG
Va-yfgM-f	CGGTACCCGGGGATCGTTTGGCCTTAACGGTCCTG
Va-yfgM-r	CGACTCTAGAGGATCTTGCCAACAATAACTGGCGC
del-Va-yfgM-f	TTAAGAGGACAGGAAGGACCGCATTGGATGAAAAG
del-Va-yfgM-r	TTCCTGTCCTCTTAAAACATG
pSW-yfgM-f	TCTGCGAGGCTGGCCGTTTGGCCTTAACGGTCCTG
pSW-yfgM-r	GATACCGTCGACGCCTTGCCAACAATAACTGGCGC

Figure S1

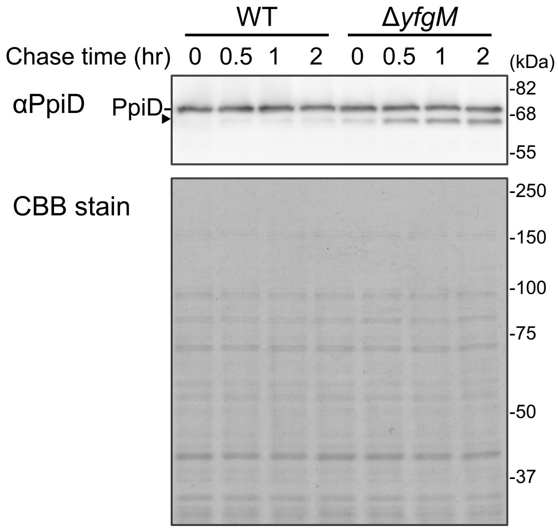

Figure S1. The stability of PpiD in the Δ*yfgM*-deficient strain. NT17 (WT) and NT35 (Δ*yfgM*) cells were grown at 30 ºC in LB medium for 2 h and spectinomycin (final concentration, 500 µg/mL) was added at time 0. After cultivation at 37 ºC for the indicated time periods, total cellular proteins were acid-precipitated and analyzed using SDS-PAGE, immunoblotting with anti-PpiD antibodies, and CBB staining. Black arrowheads indicate PpiD degradation products (PpiD').

Figure S2

A

[DNA sequence]

WT/*yfgM*⁺	AA-A/GCA/CTG(48bp)TGG/AAC/AGC
yfgM(TM f.s.)	AAG/AGC/ACT(48bp)CTG/GAA/-AGC

[Amino acid sequence]

YfgM(WT)	KALAVGVILGVGALIGWRYWNS
YfgM(TM F.S.)	KSTGCWGDFGRWRTDWLALLES

B

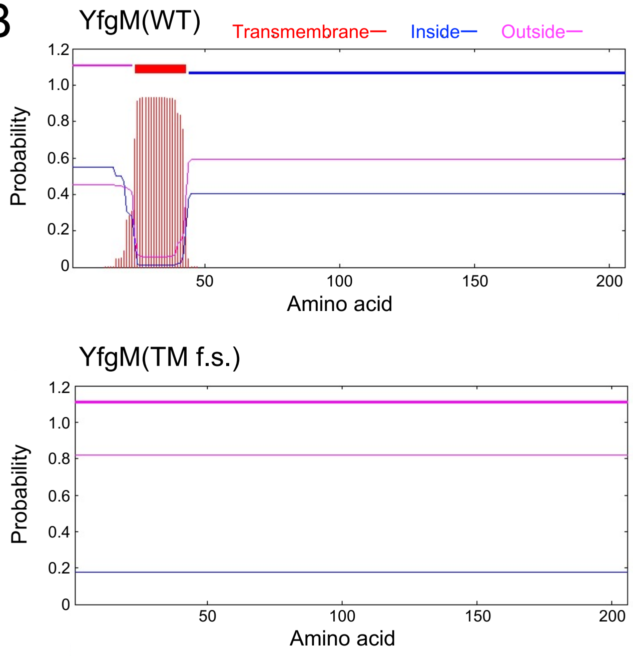

Figure S2. TM helix region in YfgM and the corresponding region in the YfgM (TM F.S.) mutant. *A*, nucleotide and amino acids sequences of the TM regions of wild-type YfgM and the corresponding region of YfgM (TM F.S.) mutant. Insertion and deletion mutations that cause a frame-shift are underlined. *B*, TM helix prediction in YfgM and the YfgM (TM F.S.) mutants. TM segments in YfgM and YfgM (TM F.S.) proteins were predicted from their amino acid sequences using the TMHMM-2.0 program (https://services.healthtech.dtu.dk/service.php?TMHMM-2.0).

Figure S3

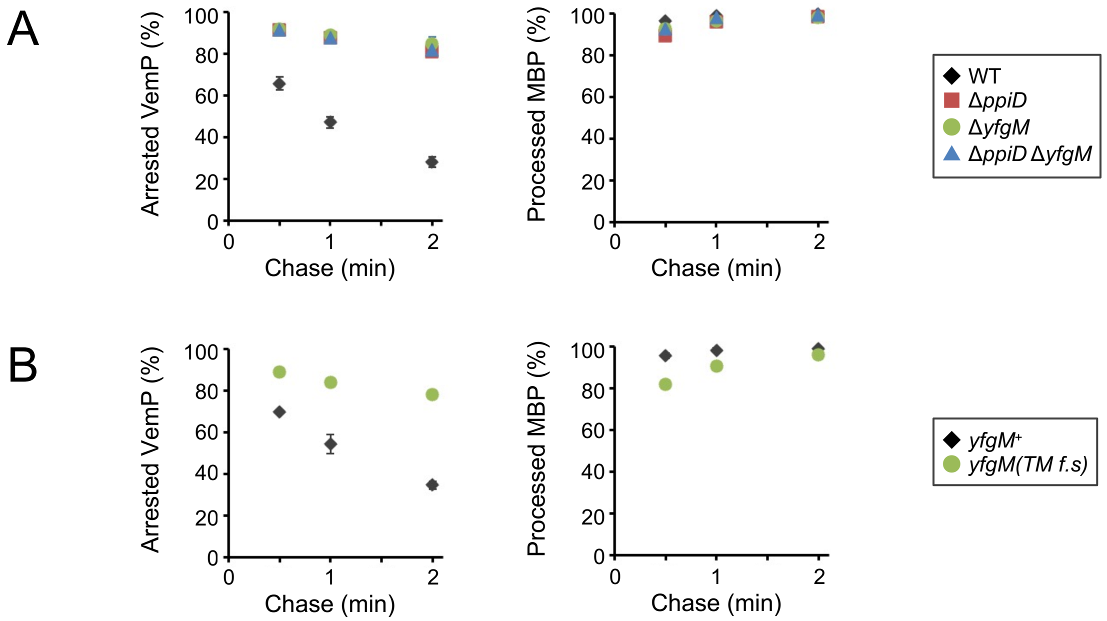

Figure S3. Stability of the arrested VemP and export ability of MBP in Δ*yfgM* and/or Δ*ppiD* mutant cells. The intensities of the VemP bands annotated as AP-unpro, AP-pro and FL mature in the upper gels and the MBP bands as precursor and mature in the lower gels of Figures 2A and 2B were quantitated and converted into relative molar amounts (RMA) using numbers of methionine residues in the species. Percentages of the arrested VemP and processed MBP were calculated by the following formulae.

Arrested VemP (%) = 100 x ([AP-unpro] + [AP-pro]) / ([FL mature] + [AP-unpro] + [AP-pro]]

Processed MBP (%) = 100 x [MBP-mature]/([MBP-mature] + [MBP-precursor])

[X] represents the RMA of the X molecules.

Average values of the arrest efficiency of VemP and the processing efficiency of MBP with S.D. ($n \geq 2$) are plotted against the chase time and shown as the left and right graphs, respectively.

Figure S4

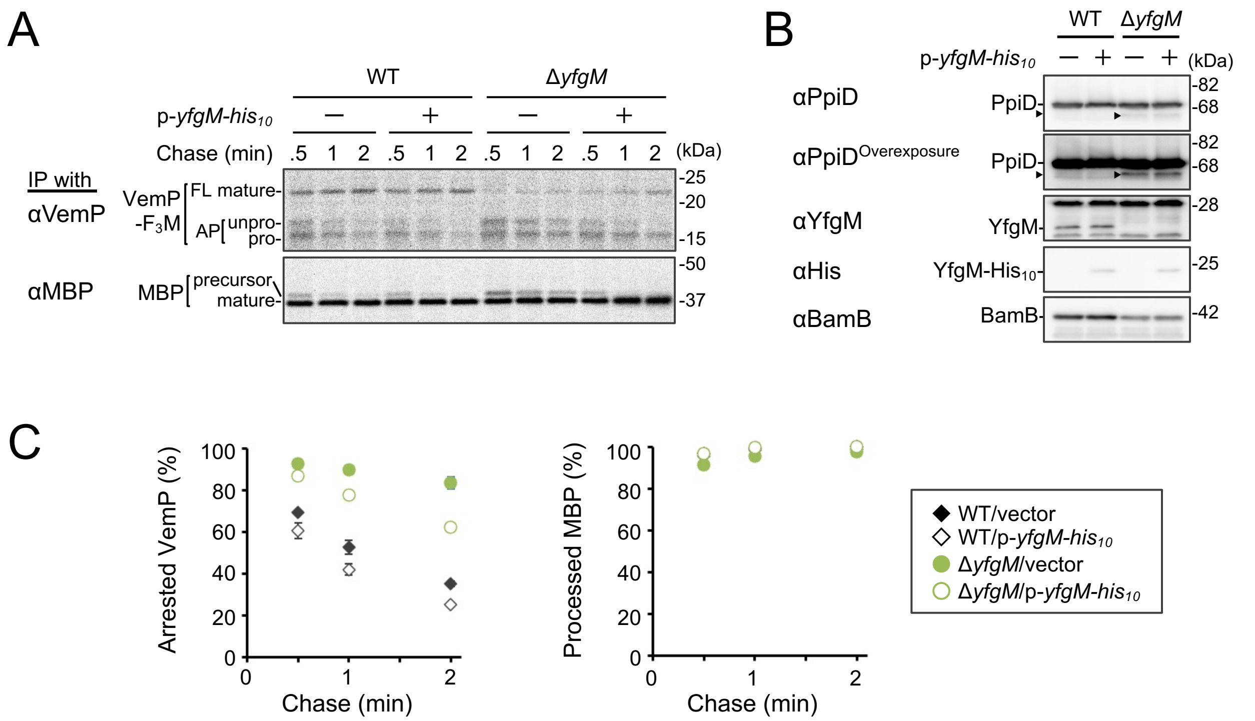

Figure S4. YfgM expression suppresses the VemP translation arrest-release defect in Δ*yfgM* cells. *A*, NT17 derived cells carrying pSTD343(*lacI^q*), pHM1021-*vemP-f$_3$m*, and either pMW118 or pMW118-*yfgM-his$_{10}$* were grown at 30 ºC in M9-based medium until early log phase, induced with 1 mM IPTG for 15 min, pulse-labeled with [^{35}S]Met for 30 s and chased for the indicated periods. At each time point, total cellular proteins were acid-precipitated, subjected to IP with anti-VemP or anti-MBP antibodies, and analyzed using SDS-PAGE followed by phosphorimaging. *B*, before radiolabeling, total cellular proteins were acid-precipitated from culture portions and analyzed using SDS-PAGE and immunoblotting with the indicated antibodies. Black arrowheads indicate PpiD degradation products (PpiD'). Some PpiD' was observed even in the Δ*yfgM* cells expressing YfgM from the plasmid (fourth lane), likely because it was derived from YfgM-free PpiD molecules stably accumulated in the Δ*yfgM* cells before the short induction (for 15 min) of YfgM. *C*, percentages of the arrested VemP and the processed MBP in *A* were calculated as in Figure S3. Average values of the arrest efficiency of VemP and the processing efficiency of MBP with S.D. (n = 2) are plotted against the chase time and shown as the left and right graphs, respectively. The short-time expression of YfgM-His$_{10}$ from the plasmid only partially suppressed the defect of the arrest-cancelation of VemP in the Δ*yfgM* cells. It is presumably due to a lower accumulation level of YfgM-His$_{10}$ (as compared with the results in Figure 1C), which was supported by the results (shown in *B*) that it was immuno-detectable with anti-His-tag antibodies (higher reactivity), but not with anti-YfgM antibodies (lower reactivity).

Figure S5

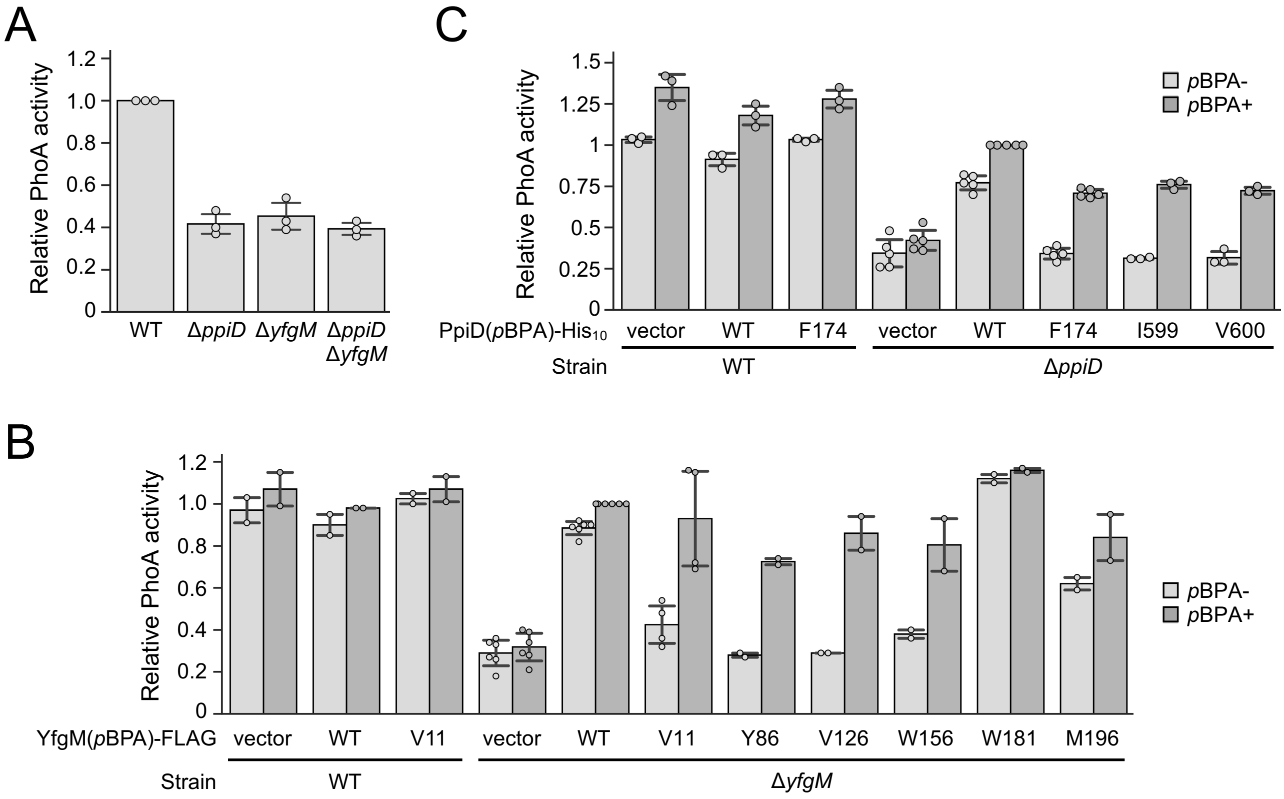

Figure S5. Monitoring of VemP-arrest cancelation using a VemP-PhoA reporter system. *A*, PhoA activity of the *yfgM* or *ppiD* mutant strain expressing VemP-PhoA reporter proteins. NT17 derivative cells carrying pHM1550-*vemP-phoA* were grown at 30 ºC in LB-medium containing 1 mM IPTG for 2.5 h to express VemP-PhoA. PhoA activity was measured according to the method described in the EXPERIMENTAL PROCEDURES. Activity is shown relative to that of the wild-type strain ($n = 3$, ± S.D.). *B*, PhoA activity of cells expressing the YfgM(*p*BPA) variants. NT17 (WT) or NT35 (Δ*yfgM*) cells carrying pEVOL-pBpF, pHM1550-*vemP-phoA*, and pTWV228-*yfgM(amb)-flag* were grown at 30 ºC in L-medium containing 1 mM IPTG with or without 0.5 mM *p*BPA for 3 h to express both the indicated YfgM(*p*BPA) variants and VemP-PhoA. PhoA activity was measured according to the method described in the EXPERIMENTAL PROCEDURES. Activity is shown relative to that of wild-type YfgM-FLAG expressing cells cultured in the presence of *p*BPA ($n \geq 2$, ± S.D.). *C*, PhoA activity of cells expressing PpiD(*p*BPA) variants. NT17 (WT) or RM3688 (Δ*ppiD*) cells carrying pEVOL-pBpF, pHM1550-*vemP-phoA*, and pHM1021-*ppiD(amb)-his_{10}* were grown at 30 ºC in LB-medium containing 1 mM IPTG and 0.02% arabinose with or without 0.5 mM *p*BPA for 3 h to express the indicated PpiD(*p*BPA) variants and VemP-PhoA. PhoA activity was measured according to the method described in the EXPERIMENTAL PROCEDURES. Activity is shown relative to that of wild-type PpiD-His_{10} expressing cells cultured in the presence of *p*BPA ($n \geq 3$, ± S.D.). All cells with plasmids carrying either *yfgM(amber)* or *ppiD(amber)* exhibited comparable PhoA activity to wild type *yfgM* or *ppiD* plasmid-harboring cells when cultivated in medium containing *p*BPA, indicating that all YfgM(*p*BPA) and PpiD(*p*BPA) derivatives were functional. The PhoA activities of cells expressing either YfgM(*p*BPA) and PpiD(*p*BPA) derivatives were comparable to those of the wild type cells with or without the expression of either wild type PpiD, wild type YfgM or their *p*BPA derivatives, strongly suggesting that only the PpiD/YfgM complex can mediate the TTA of VemP-PhoA, and the presence of an excess amount of free (un-complexed) YfgM or PpiD little affects it. Cells with a plasmid carrying either *yfgM(W181amber)* or *yfgM(M196amber)* had significantly higher PhoA activity, even in the absence of *p*BPA, compared to cells carrying the other *yfgM(amber)*, suggesting that small C-terminal region truncations do not dramatically reduce YfgM's activity.

Figure S6

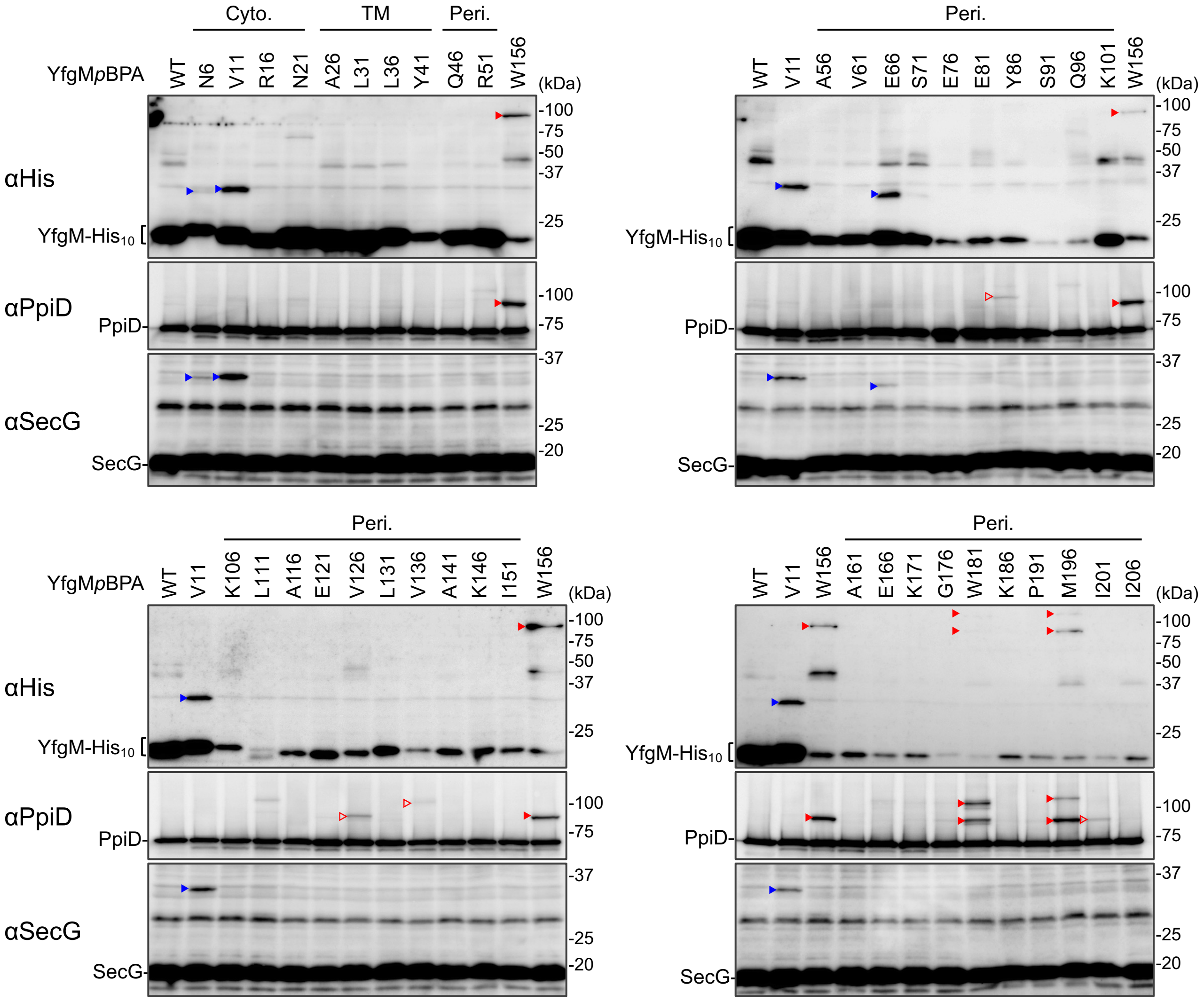

Figure S6. Systematic *in vivo* photo-crosslinking analysis of YfgM. NT35 (Δ*yfgM*) cells carrying both pEVOL-pBpF and pMW118-*yfgM(amb)-his$_{10}$* were grown at 30 ºC in L-medium containing 0.5 mM *p*BPA until early log phase and induced with 1 mM IPTG for 1 h to express the indicated YfgM(*p*BPA) variants. After UV-irradiation for 10 min at 4 ºC, total cellular proteins were acid-precipitated and analyzed using SDS-PAGE and immunoblotting with the indicated antibodies. YfgM-PpiD and YfgM-SecG crosslinked products are shown as red and blue arrowheads, respectively. YfgM-PpiD crosslinked products detected using both anti-His tag and anti-PpiD antibodies and those detected using only anti-PpiD antibodies are represented by closed and open arrowheads, respectively.

Figure S7

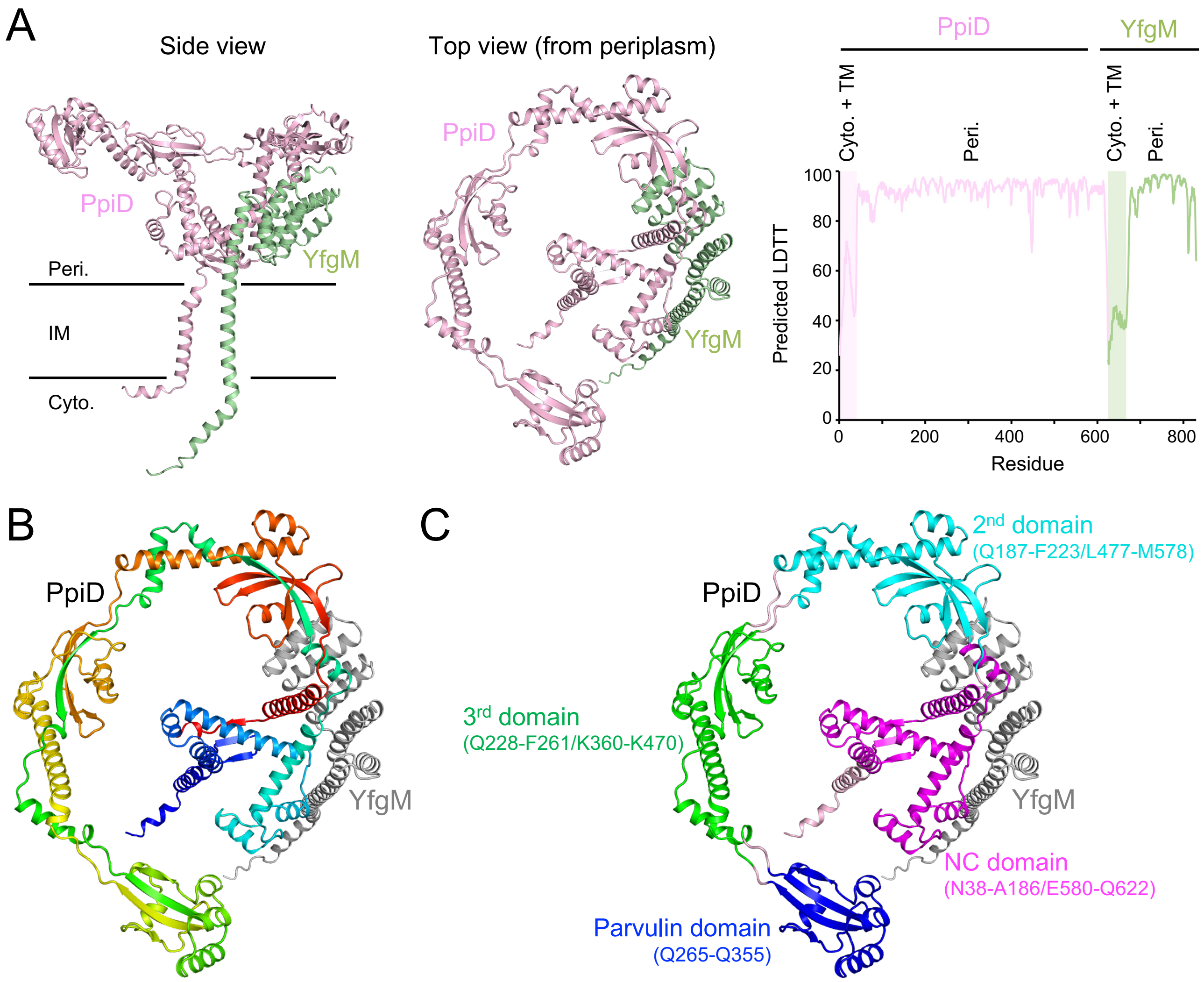

Figure S7. Structural model of the PpiD/YfgM heterodimer predicted using AlphaFold2. *A*, ribbon representation of the predicted structure of an *E. coli* PpiD/YfgM heterodimer. PpiD and YfgM are indicated in pink and pale green, respectively. A local distance difference test (LDDT) of the predicted PpiD/YfgM heterodimer model is plotted against the YfgM and PpiD sequences. Horizontal axis: consecutive residue numbers in which the PpiD amino acid sequence is followed by that of YfgM. *B*, rainbow representation of the PpiD molecule in the complex. Colors from blue to red indicate the topology from the N- to the C-terminus. YfgM is indicated in gray. *C*, domain structures of PpiD in the complex. The periplasmic region, NC domain, 2nd domain, 3rd domain, and Parvulin domain are indicated in magenta, cyan, green, and blue, respectively.

Figure S8

A

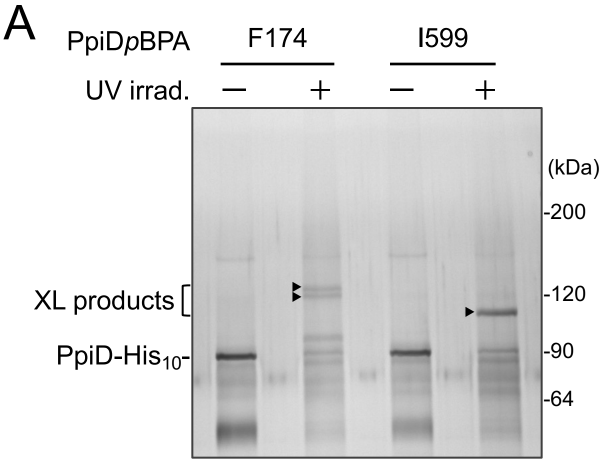

PpiD F174*p*BPA(upper)

Description	Abundance
PpiD	838,763,892
YfgM	176,325,936
SlyD	4,875,270
AceF	2,660,722
ZntA	1,004,830

PpiD I599*p*BPA

Description	Abundance
PpiD	1,883,123,659
YfgM	361,977,699
SlyD	2,991,508
AceF	1,511,034
RbsD	524,180

PpiD F174*p*BPA(lower)

Description	Abundance
PpiD	470,114,768
YfgM	99,491,626
ZntA	9,994,311
SlyD	7,078,184
AceF	1,212,496

B

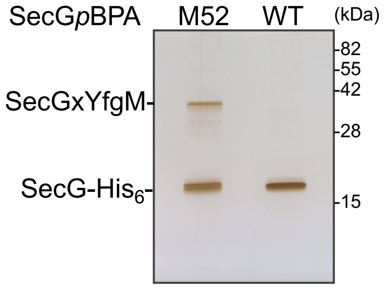

SecG M52*p*BPA

Description	emPAI
YfgM	5.75
SecG	0.35

Figure S8. MS analyses of the crosslinked products. *A*, PpiD-SecG crosslinked products. RM3688 (Δ*ppiD*) cells carrying pEVOL-pBpF and either pHM1021-*ppiD(F174amb)-his*$_{10}$ or pHM1021-*ppiD(I599amb)-his*$_{10}$ were grown at 30 ºC in LB-medium supplemented with 0.02% arabinose and 0.5 mM *p*BPA until early-log phase and induced with 1 mM IPTG for 1 h. Crosslinked products and PpiD-His$_{10}$ were purified, separated using SDS-PAGE, and analyzed according to the method described in the EXPERIMENTAL PROCEDURES. *B*, YfgM-SecG crosslinked products. SA101 (Δ*secG*) cells carrying pHM649 and either pMW118-*secG(M52amb)-his*$_6$ or pMW118-*secG-his*$_6$ were grown at 30 ºC in the M9 based medium supplemented with 1 mM *p*BPA until mid-log phase. Crosslinked products and SecG-His$_6$ were purified, separated using SDS-PAGE, and analyzed according to the method described in the EXPERIMENTAL PROCEDURES.

Figure S9

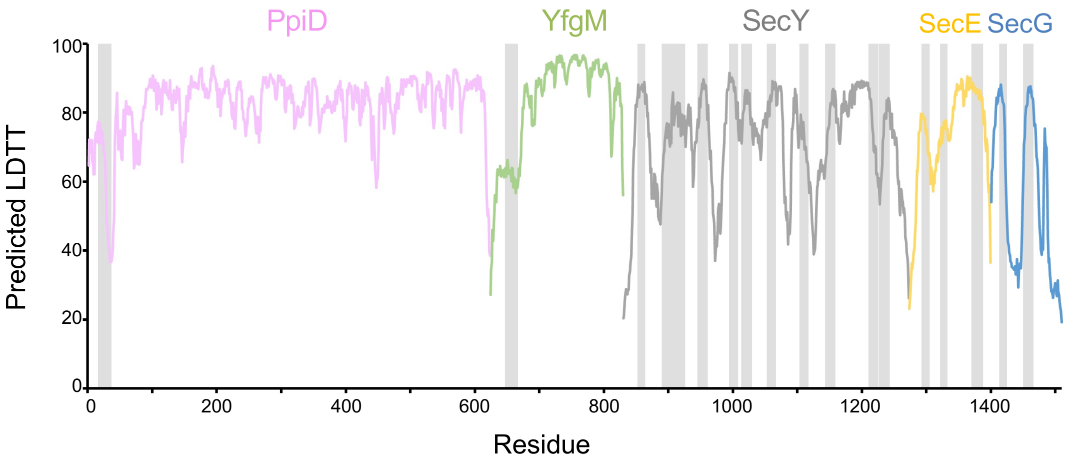

Figure S9. Local distance difference test (LDDT) of the predicted PpiD/YfgM-SecY/E/G super-complex. The LDDT of the predicted model of the PpiD/YfgM-SecYEG super complex is plotted against the amino acid residue positions along the PpiD, YfgM, SecY, SecE, and SecG sequences. Horizontal axis: consecutive residue numbers of the PpiD amino acid sequence followed by those of YfgM, SecY, SecE, and SecG. Regions corresponding to the TM segments of these proteins are shown as gray bars. Since the LDDT values of the regions in the model corresponding to all SecY TM segments, TM3 of SecE (functionally essential), and the two TMs of SecG were high, the membrane-embedded core structure of SecY/E/G appears sufficiently reliable.

Figure S10

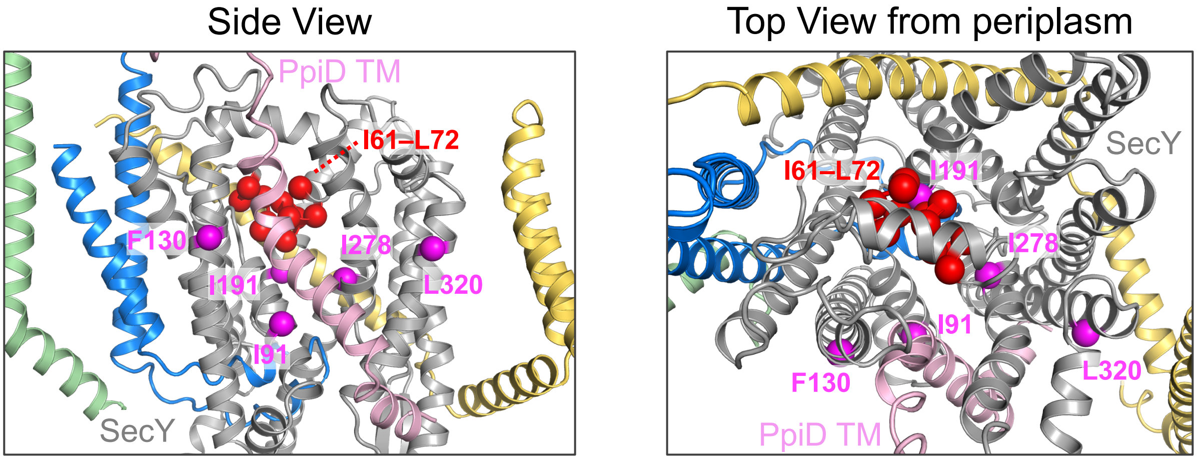

Figure S10. Close-up views of PpiD-crosslinking sites in SecY of the predicted PpiD/YfgM-SecY/E/G super-complex. The PpiD-neighbor residues in the TM regions (magenta) and the plug helix (red) of SecY are mapped on the model structure, and viewed from the front side of SecY/E/G (left) and the periplasmic side (right). SecY, SecE, SecG, PpiD and YfgM are colored in gray, yellow, blue, pink, and pale green, respectively.

Figure S11

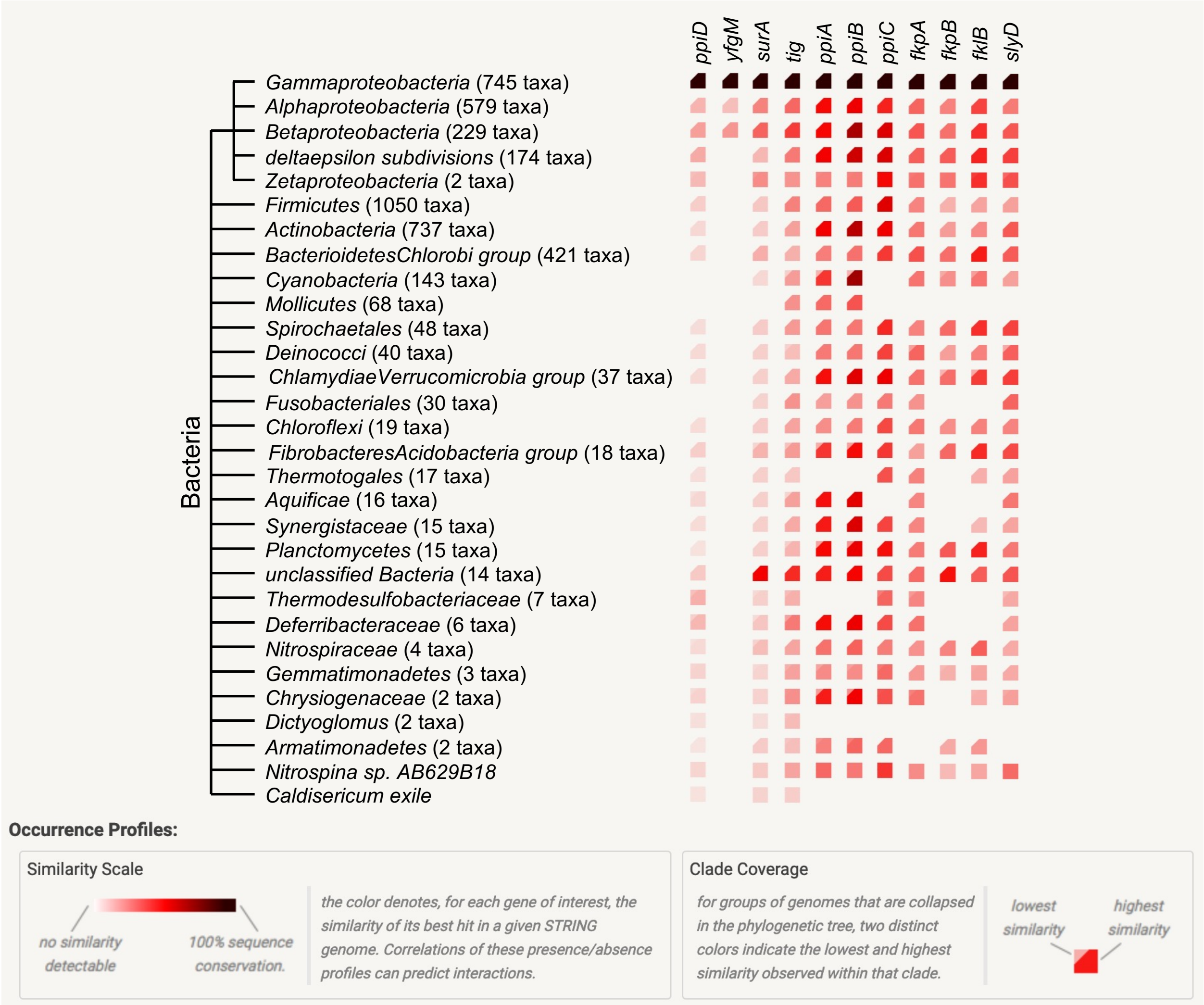

Figure S11. Gene co-occurrence of *E. coli ppiD*, *yfgM,* and other PPIase genes. Gene co-occurrence analysis for *E. coli ppiD*, *yfgM,* and other PPIases (*surA*, *tig*, *ppiA*, *ppiB*, *ppiC*, *fkpA*, *fkpB*, *fklB,* and *slyD*) was conducted using the STRING Database (https://string-db.org/).
